# Similar Responses of Relatively Salt-Tolerant Plants to Na and K during Chloride Salinity: Comparison of Growth, Water Content and Ion Accumulation

**DOI:** 10.3390/life12101577

**Published:** 2022-10-11

**Authors:** Gederts Ievinsh, Una Andersone-Ozola, Astra Jēkabsone

**Affiliations:** Department of Plant Physiology, Faculty of Biology, University of Latvia, 1 Jelgavas Street, LV-1004 Riga, Latvia

**Keywords:** electrical conductivity, halophytes, ion accumulation, potassium, salinity, sodium, water content

## Abstract

The aim of the present study was to compare changes in growth, ion accumulation and tissue water content in relatively salt-tolerant plant taxa—*Beta vulgaris* subsp. *maritima*, *Beta vulgaris* subsp. *vulgaris* var. *cicla*, *Cochlearia officinalis*, *Mentha aquatica* and *Plantago maritima*—as a result of NaCl and KCl salinity in controlled conditions. Similar growth responses to Na^+^ and K^+^ salinity in a form of chloride salts were found for all model plants, including growth stimulation at low concentrations, an increase in water content in leaves, and growth inhibition at high salinity for less salt-resistant taxa. All plant taxa were cultivated in soil except *M. aquatica*, which was cultivated in hydroponics. While the morphological responses of *B. vulgaris* subsp. *vulgaris* var. *cicla*, *B. vulgaris* subsp. *maritima* and *P. maritima* plants to NaCl and KCl were rather similar, *C. officinalis* plants tended to perform worse when treated with KCl, but the opposite was evident for *M. aquatica*. Plants treated with KCl accumulated higher concentrations of K^+^ in comparison to the accumulation of Na^+^ in plants treated with equimolar concentrations of NaCl. KCl-treated plants also had higher tissue levels of electrical conductivity than NaCl-treated plants. Based on the results of the present study, it seems that both positive and negative effects of Na^+^ and K^+^ on plant growth were due to unspecific ionic effects of monovalent cations or/and the specific effect of Cl^−^.

## 1. Introduction

Soil salinity and plant salt tolerance has been an object of intense scientific studies for many decades. The scientific understanding of these processes has consequently improved significantly, as illustrated by the recent reviews [1,2,3,4,5,6]. Since both Na^+^ and Cl^−^ are the most abundant elements in seawater, it makes sense that the majority of studies specifically use NaCl to assess the effect of salinity. However, this uniformity somewhat limits our ability to understand the diversity of plant salinity responses. Practically, there are soils where other ions besides Na^+^ and Cl^+^ also significantly participate in the formation of salinity [7]. Theoretically, the response of plants to other cations such as K^+^ may provide an opportunity to understand Na^+^ response mechanisms due to the chemical similarity of the two elements.

Na^+^ toxicity is traditionally considered as one of the main reasons for plant growth inhibition by NaCl [8]. However, while based on practical evidence on negative effects of salinity, this thought is somewhat of a simplification, as from a chemical point of view, Na^+^ is very similar to K^+^ and therefore cannot be “more toxic” by definition. However, unlike K^+^, Na^+^ is not an essential element for most plants, and limiting its uptake or promoting its storage in plant tissues could have significant physiological costs. Therefore, there is a reason to believe that for halophytes, an ability to use Na^+^ instead of K^+^ for some of its functions would confer certain adaptive advantages [9]. There is no doubt that Na^+^ has characteristically more negative effects than that of K^+^ for typical glycophyte species [10]. However, while some studies have shown that the effect of NaCl and KCl in certain obligate halophytic plant species is similar, such as *Atriplex nummularia* [11], *Sesuvium portulacastrum* [12] and *Atriplex halimus* [13], there is still no conclusive confirmation of the functional difference or similarity between the two elements in various salt-tolerant plant taxa.

The control of the compartmentation of osmotically and electrolytically active ions is one of the most important aspects of plant tolerance to salinity [14]. Some salt-tolerant species use ion exclusion strategy, not allowing for a buildup of a high salt concentration in aboveground tissues [4]. However, the majority of halophytes are thought to represent salt accumulators, but even for these species, the ion concentration in the mesophyll tissues of photosynthetically active organs can be diminished by means of salt secretion to the leaf surface (recretohalophytes), an increase in tissue water content (succulent halophytes) and the accumulation of salts in older leaves [4]. Salt-accumulating halophytes use a cellular compartmentation strategy, accumulating salts in vacuoles, further solving a problem of proper osmotic adjustment [15]. The control of electrolytical activity in plant tissues due to salinity has been much less often considered, but it seems that coastal species from salt-affected habitats show different strategies, regulating leaf tissue electrical conductivity (EC) by changes in either K^+^ or Na^+^ concentration, but some species relatively tightly control the EC level by concomitant changes in both K^+^ and Na^+^ concentration [16].

To comparatively assess the effect of NaCl and KCl on growth and ion accumulation, five model plant taxa from four families with predictably good salinity tolerance were selected for the study. *Beta vulgaris* subsp. *maritima* (L.) Arcang. (Amaranthaceae) is a coastal-specific plant taxon, growing on sandy, pebbly or rocky beaches, and it is considered to represent the wild ancestor of all beet crops [17]. The taxon is widely distributed around Europe and the Mediterranean Sea [18]. It is a characteristic plant of the European protected habitats 1230 “Vegetated sea cliffs of the Atlantic and Baltic coasts” and 1330 “Atlantic salt meadows (Glauco-Puccinellietalia maritimae)” [19]. Among cultivated beets, the leafy beet type Swiss chard has retained a morphology that is in general similar to that of *B. vulgaris* subsp. *maritima* [20]. It is classified as *Beta vulgaris* L. subsp. *vulgaris* var. *cicla*. The salinity tolerance of both *B. vulgaris* subsp. *maritima* [21,22,23,24] and *B. vulgaris* subsp. *vulgaris* var. *cicla* [25,26,27] has been assessed previously. In general, it was concluded that both are typical halophytic taxa, and the domestication process rather slightly reduced the salinity tolerance of beet crops [28].

The genus *Cochlearia* (Brassicaceae) is considered to be represented only by extremophile species, being halophytes or metallophytes [29]. Among them, *Cochlearia officinalis* L. is a potential saline vegetable crop [30]. *C. officinalis* is regarded as a typical halophyte usually found in brackish conditions of coastal areas, on gravel beaches and dry areas of saltmarshes, restricted to the northern hemisphere [31]. It is a characteristic species of the European habitat 1230 “Vegetated sea cliffs of the Atlantic and Baltic coasts” [32]. However, the species has been classified as only moderately salinity tolerant [30].

*Mentha aquatica* L. (Lamiaceae) is a clonal species well-adapted to flooded conditions; it forms long runners both from above-ground and below-ground parts of the stem, with an ability to produce several ramet shoots from a single individual [33]. Biomass accumulation in *M. aquatica* is significantly stimulated by increasing soil moisture and, especially, soil waterlogging [34]. The species is often found in river streams and shallow waters of lakes. It is a characteristic species of the European protected habitats 3260 “Water courses of plain to montane levels with the Ranunculion fluitantis and Callitricho-Batrachion vegetation” [35] and 6430 “Hydrophyllous tall herb fringe communities of plains and the montane to alpine levels” [36]. The *M. aquatica* population from a seawater-affected coastal habitat has been identified recently [16], and these plants showed a prominent potential for use in hydroponic-based biological air purification systems, facilitating the development of beneficial microbiome [37]. So far, only a single study assessed salinity responses in *M. aquatica,* and it was characterized as moderately tolerant [34].

*Plantago maritima* L. (Plantaginaceae) is a halophytic species found on European coastal habitats 1230 “Vegetated sea cliffs of the Atlantic and Baltic coasts”, 1330 “Atlantic salt meadows (Glauco-Puccinellietalia maritimae)” and 1630 “Boreal Baltic coastal meadows”, as well as on inland habitats 1340 “Inland salt meadows” and 1530 “Pannonic salt steppes and salt marshes” [32]. Earlier studies have established the high salinity tolerance and halophytic nature of *P. maritima* [38,39,40]. High phenotypic plasticity and the life-cycle-stage-dependence of responses to abiotic factors were noted for the species [41]. However, as based on a more recent study, *P. maritima* has been designated as a facultative halophyte [42]. Using *P. maritima* as a model plant, it has been shown that NaCl interacts with nitrate transporters in the plasma membrane of roots cells, decreasing the rate of nitrate uptake in saline soil [43]. The species has also been used in ecological studies, showing that competition for light and flooding are among important factors that affect the demographics of *P. maritima* in coastal meadows [44,45,46].

Thus, the aim of the present study was to compare changes in growth, ion accumulation and tissue water content in relatively salt-tolerant plant taxa—*Beta vulgaris* subsp. *maritima*, *Beta vulgaris* subsp. *vulgaris* var. *cicla*, *Cochlearia officinalis*, *Mentha aquatica* and *Plantago maritima*—in controlled conditions due to NaCl and KCl salinity. It was hypothesized that both cations in a form of chloride salt will have similar effects on the selected model plants.

## 2. Materials and Methods

### 2.1. Plant Material

Five taxa were used as model plants in the present study (Table 1). Plants of *Beta* *vulgaris* subsp. *vulgaris* var. *cicla* cv. ‘Magenta Sunset’ (BVC), *Beta vulgaris* subsp. *maritima* (BVM), *Cochlearia officinalis* (CO) and *Plantago maritima* (PM) were established from seed and cultivated in soil-like substrate. Plants of *Mentha aquatica* (MA) were established from stem explants collected from emergent plants on a seawater-affected sandy beach habitat and further cultivated in hydroponics.

### 2.2. Plant Establishment, Cultivation and Treatments

Before germination, seeds were surface-sterilized with 50% commercial bleach Ace (Procter & Gamble, Warszawa, Poland) for 7 min followed by washing in sterile deionized water (10 × 2 min). Seeds were imbibed in sterile deionized water for 4 h and sown in sterile plastic tissue culture containers with 1 cm of autoclaved commercial garden soil (Biolan, Eura, Finland) mixed with sterile deionized water. Containers were placed in a plant growth cabinet MLR-352H (Sanyo Electric, Osaka, Japan) with a photoperiod of 16 h (40 µmol m^−2^ s^−1^) and day/night temperatures of 20/15 °C. Seedlings were transplanted to 200 mL plastic containers filled with a mixture of quartz sand (Saulkalne S, Saulkalne, Latvia) and heat-treated (60 °C, 24 h) garden soil (Biolan, Eura, Finland) 1:4 (*v*/*v*) after the appearance of the first two true leaves. Containers were placed in 48 L plastic boxes closed with lids, placed in a greenhouse and gradually adapted to greenhouse conditions. An experimental automated greenhouse (HortiMaX, Maasdijk, Netherlands) was used for the study. Supplemented light was provided by Master SON-TPIA Green Power CG T 400 W (Philips, Amsterdam, Netherlands) and Powerstar HQI-BT 400 W/D PRO (Osram, Munich, Germany) lamps (photon flux density of photosynthetically active radiation 380 µmol m^−2^ s^−1^ at the plant level), with a 16 h photoperiod. The day/night temperature was 23/16 °C, and the relative air humidity was maintained at 60 to 70%. When plants reached 5 to 10 cm heights, they were transplanted to 1.2 L plastic containers filled with a 1 L mixture of quartz sand (Saulkalne S, Saulkalne, Latvia) and garden soil (Biolan, Eura, Finland) in different proportions (Table 1). Soil was moistened with deionized water. The substrate water content was monitored with an HH2 moisture meter equipped with a WET-2 sensor (Delta-T Devices, UK) and maintained at no less than 50% throughout the experiment using deionized water. Individual containers were randomly placed on a greenhouse bench and repositioned once a week. Every week, plants were fertilized with Yara Tera Kristalon Red and Yara Tera Calcinit fertilizers (Yara International, Oslo, Norway). A stock solution was prepared for each fertilizer (100 g L^−1^), and the working solution contained 25 mL of each per 10 L of deionized water, used with a rate of 100 mL per container.

Salinity treatment for soil-grown plants was started after a week-long period of additional acclimatization in the greenhouse. Individual plants were randomly distributed for treatments, with five plants per treatment. The salt treatment was performed gradually, twice a week, in 44 mmol L^−1^ increments during 5 weeks, using NaCl and KCl solution. A necessary amount of salt was dissolved in deionized water, and 100 mL per container was applied to soil. The total treatment doses of NaCl and KCl are given in Table 1. Control plants received deionized water.

Plants of *M. aquatica* were propagated with stem explants derived from a stock culture kept in a greenhouse. Explants were rooted in deionized water, and uniform plants with newly developed healthy shoots and well-developed root systems were transferred to 1 L hydroponic vessels, with three individual plants per vessel and three vessels per treatment. Yara Tera Kristalon Red and Yara Tera Calcinit fertilizers (1:1 *w*/*w*, 0.5 g L^−1^) were used as fertilizer medium to which an appropriate amount of NaCl and KCl was added. The salt concentration was increased and added gradually, 25 mmol L^−1^ per week, to reach the necessary concentration (25, 50, 100 and 200 mmol L^−1^). Fertilizer solution was used for control plants. The fertilizer and respective salt solution was changed weekly. Plants were cultivated in a growth cabinet with a photon flux density of photosynthetically active radiation 100 µmol m^−2^ s^−1^ at the plant level, with a 16 h photoperiod. The day/night temperature was 22/16 °C, and the relative air humidity was maintained at 60 to 70%.

### 2.3. Plant Harvest and Measurements

Experiments were terminated 4–7 weeks after reaching full treatment according to the individual growth characteristics of model plants (after 4 weeks for *B. vulgaris* subsp. *vulgaris* var. *cicla* and *C. officinalis*, 5 weeks for *B. vulgaris* subsp. *maritima*, 6 weeks for *M. aquatica* and 7 weeks for *P. maritima*). Soil EC was measured at the end of the *P. maritima* experiment using an HH2 meter equipped with a WET-2 sensor (Delta-T Devices, Burwell, UK) at four sides of each container. For *B. vulgaris* subsp. *vulgaris* var. *cicla*, *B. vulgaris* subsp. *maritima* and *P. maritima* plants, the leaves were separated according to their age and position, as well as size, in senescent, old, middle and young leaves. For both *B. vulgaris* taxa, leaf petioles and leaf blades were handled separately. For *P. maritima*, flower stalks were counted, their lengths were measured, and inflorescences and stalks were handled separately. For *C. officinalis*, leaves were separated in leaf petioles and leaf blades. For *M. aquatica*, plants were separated in the roots, stems and leaves, and the stem length was measured. All leaves were counted. Plant roots were separated from substrate and carefully washed to remove any soil particles. All plant material was weighed before and after drying in an oven at 60 °C for 72 h. The tissue water content was estimated as a mass of water in grams per gram of dry mass of tissues.

For the estimation of Na^+^, K^+^ and EC, plant tissues were crushed by hand to small pieces, and a sample of 0.2 g was randomly taken from the total amount of leaf material. Tissues were ground with a mortar and pestle to a fine powder, and 10 mL of deionized water was added. The homogenate was stirred with a pestle for 1 min. After filtration through nylon mesh cloth (No. 80), the homogenate was used for the measurement of ion concentration using LAQUAtwin compact meters B-722 (Na^+^) and B-731 (K^+^), and electrical conductivity using a LAQUAtwin conductivity meter B-771 (Horiba Scientific, Kyoto, Japan). Three individual samples per treatment of particular plant parts of particular species were analyzed in three analytical replicates.

### 2.4. Data Analysis

The results were analyzed via KaleidaGraph (v. 5.0, Synergy Software, Reading, PA, USA). The statistical significance of the differences was evaluated with one-way ANOVA using post hoc analysis with minimum significant difference. Significant differences were indicated at *p* < 0.05.

## 3. Results

### 3.1. Effect on Plant Growth and Water Content

**Figure 1:**
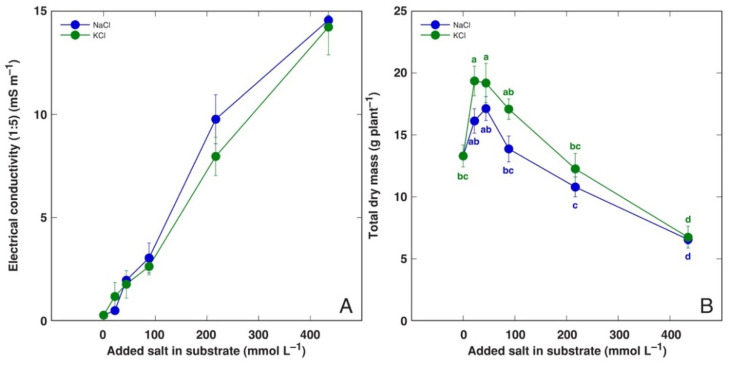
Substrate electrical conductivity (EC_1:5_) after cultivation of *Plantago maritima* plants treated with different doses of NaCl and KCl (**A**) and effect of treatments on total dry biomass of *P. maritima* plants (**B**). Different letters indicate statistically significant (*p* < 0.05) differences between treatments.

Treatment with equimolar concentrations of NaCl and KCl resulted in identical increases in substrate salinity, as seen in the experiment with *P. maritima* (Figure 1A). The morphological appearance of *P. maritima* plants treated with NaCl or KCl was similar at all salinity levels (Figure S1). Total biomass accumulation in *P. maritima* plants was stimulated at 22 and 44 mmol L^−1^ salinity, but only for KCl was this effect statistically significant (Figure 1B). An increase in salinity resulted in a decrease in plant biomass, but only plants grown at 434 mmol L^−1^ had significantly lower biomasses in comparison to that in the control. Detailed information on the morphological effects of salinity on *P. maritima* plants are given in Table S1. Most importantly, the biomass increase at low salinity was associated with an increase in the number and biomass of leaves, the number and biomass of inflorescences, as well as the biomass of roots.

**Figure 2:**
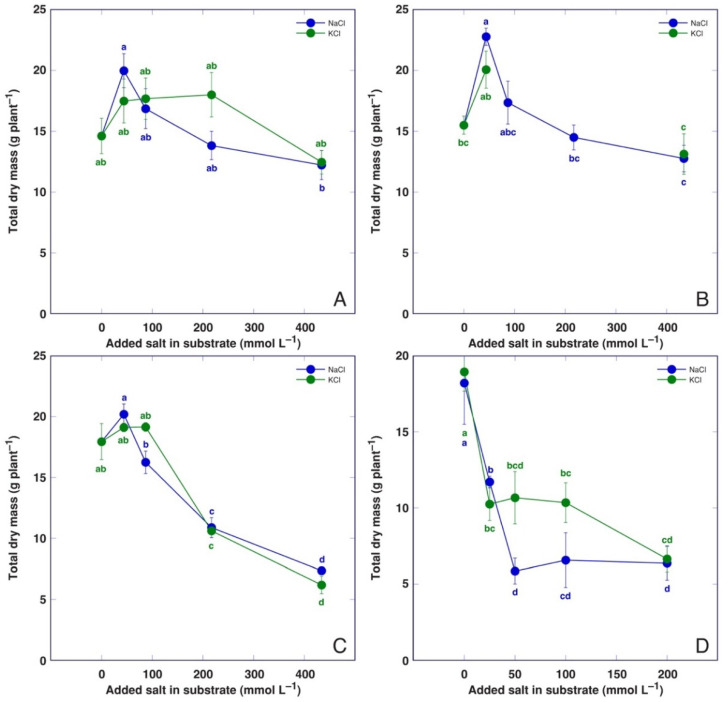
Effect of treatment with different doses of NaCl and KCl on total dry biomass of *Beta vulgaris* subsp. *vulgaris* var. *cicla* (**A**), *Beta vulgaris* subsp. *maritima* (**B**), *Cochlearia offficinalis* (**C**) and *Mentha aquatica* (**D**) plants. Different letters indicate statistically significant (*p* < 0.05) differences between treatments.

The stimulated elongation of leaf petioles was a characteristic morphological response of BVC plants at increased substrate salinity (Figure S2), but the total biomass was not significantly affected by the treatment at any concentration both for NaCl and KCl (Figure 2A). However, there was a significant difference in biomass between BVC plants treated with 44 and 434 mmol L^−1^ NaCl. Detailed information on the morphological effects of salinity on BVC plants are given in Table S2.

At low salinity, BVM plants also showed the stimulated elongation of leaf petioles (Figure S3), but a significant increase in total biomass was only evident for plants treated with 44 mmol L^−1^ NaCl (Figure 2B). No significant decrease in biomass was evident with increasing substrate salinity in comparison to the control. Detailed information regarding the morphological effects of salinity on BVM plants is given in Table S3. A biomass increase at low NaCl was associated with an increase in the number and biomass of leaves, as well as the biomass of roots.

*C. maritima* plants showed a typical decrease in leaf size with increasing salinity (Figure S4). The total biomass significantly decreased at 217 mmol L^−1^ for both NaCl- and KCl-treated plants, and further decreased at 434 mmol L^−1^ (Figure 2C). Detailed information on the morphological effects of salinity on *C. maritima* plants are given in Table S4. The number of leaves, leaf and root biomass were strongly negatively affected by increasing salinity for both NaCl- and KCl-treated plants.

The total biomass of *M. aquatica* plants significantly decreased already at 44 mmol L^−1^ salinity, and this effect was more pronounced for NaCl-treated plants (Figure 2D). However, the negative effect did not further increase with the increasing salinity of the cultivation medium. Detailed information on the morphological effects of salinity on *M. aquatica* plants is given in Table S5.

**Figure 3:**
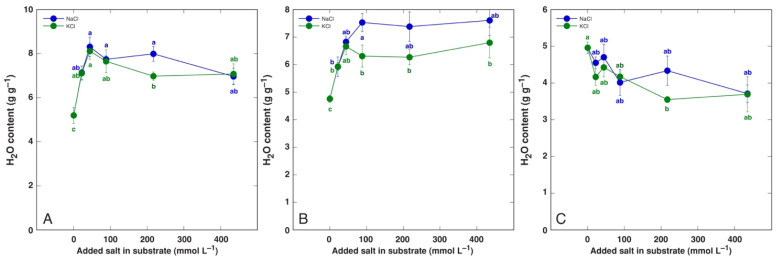
Tissue water content in old leaves (**A**), young leaves (**B**) and roots (**C**) of *Plantago maritima* plants treated with different doses of NaCl and KCl. Different letters indicate statistically significant (*p* < 0.05) differences between treatments.

**Figure 4:**
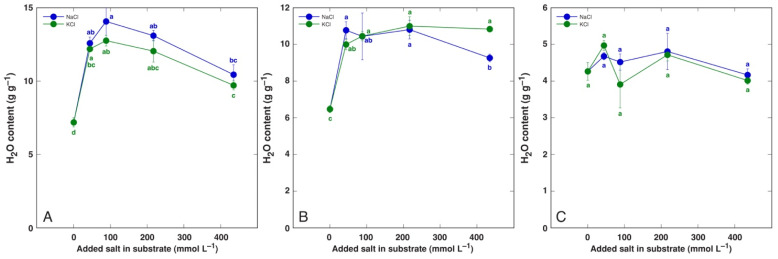
Tissue water content in old leaves (**A**), young leaves (**B**) and roots (**C**) of *Beta vulgaris* subsp. *vulgaris* var. *cicla* plants treated with different doses of NaCl and KCl. Different letters indicate statistically significant (*p* < 0.05) differences between treatments.

**Figure 5:**
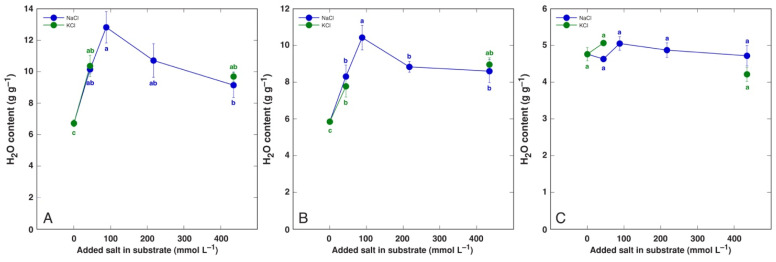
Tissue water content in old leaves (**A**), young leaves (**B**) and roots (**C**) of *Beta maritima* subsp. *maritima* plants treated with different doses of NaCl and KCl. Different letters indicate statistically significant (*p* < 0.05) differences between treatments.

**Figure 6:**
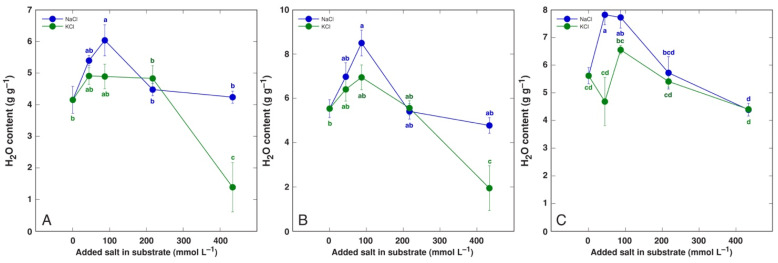
Tissue water content leaf petioles (**A**), leaf blades (**B**) and roots (**C**) of *Cochlearia officinalis* plants treated with different doses of NaCl and KCl. Different letters indicate statistically significant (*p* < 0.05) differences between treatments.

**Figure 7:**
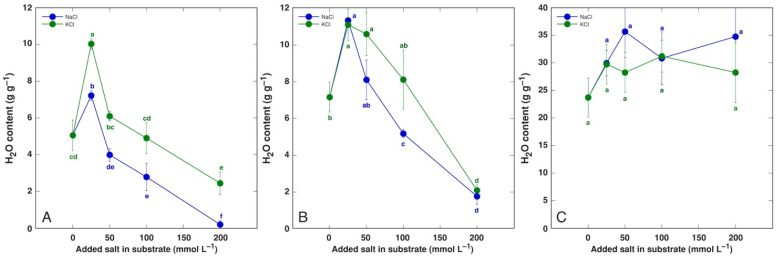
Tissue water content in leaves (**A**), stems (**B**) and roots (**C**) of *Mentha aquatica* plants treated with different doses of NaCl and KCl. Different letters indicate statistically significant (*p* < 0.05) differences between treatments.

A significant increase in tissue water content by NaCl and KCl treatment at all concentrations was evident in both old and young leaves of *P. maritima* (Figure 3), BVC (Figure 4) and BVM (Figure 5) plants. In contrast, no changes in root water content were evident for these model plants. For *C. officinalis*, a significant increase in tissue water content was seen only in the leaf blades and petioles of plants treated with 87 mmol L^−1^ NaCl and the roots of both 44 and 87 mmol L^−1^ NaCl-treated plants (Figure 6). However, a significant decrease in water content was evident in the leaf blades and petioles of plants treated with 434 mmol L^−1^ KCl. Both the leaves and stems of *M. aquatica* plants grown at 25 mmol L^−1^ NaCl and KCl showed increased water content (Figure 7). Plants treated with 100 and 200 mmol L^−1^ NaCl and 200 mmol L^−1^ KCl had significantly decreased water content in both leaves and stems. However, no significant changes in root water content were evident.

### 3.2. Effect on Ion Accumulation

**Figure 8:**
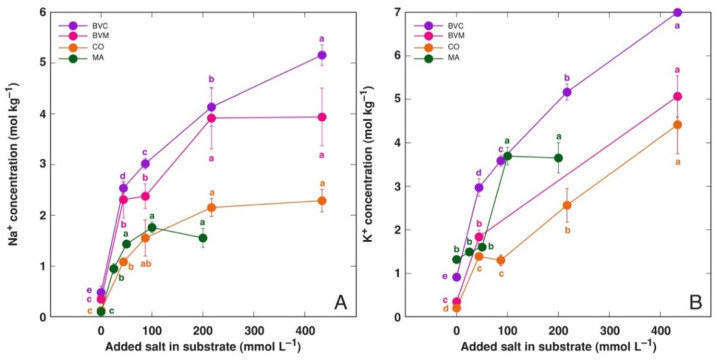
Effect of treatment with different doses of NaCl on Na^+^ concentration in leaves (**A**) and effect of treatment with different doses of KCl on K^+^ concentration in leaves (**B**) of *Beta vulgaris* subsp. *vulgaris* var. *cicla* (BVC), *Beta vulgaris* subsp. *maritima* (BVM), *Cochlearia officinalis* (CO) and *Mentha aquatica* (MA) plants. Different letters of respective colors indicate statistically significant (*p* < 0.05) differences between treatments for a respective plant taxon.

**Figure 9:**
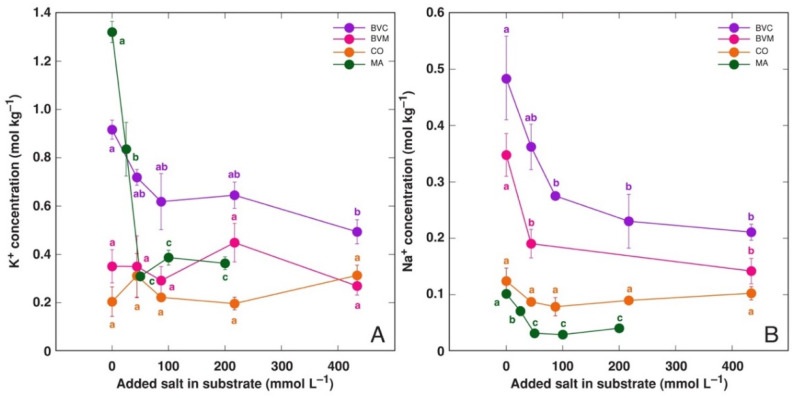
Effect of treatment with different doses of NaCl on K^+^ concentration in leaves (**A**) and effect of treatment with different doses of KCl on Na^+^ concentration in leaves (**B**) of *Beta vulgaris* subsp. *vulgaris* var. *cicla* (BVC), *Beta vulgaris* subsp. *maritima* (BVM), *Cochlearia officinalis* (CO) and *Mentha aquatica* (MA) plants. Different letters of respective colors indicate statistically significant (*p* < 0.05) differences between treatments for a respective plant taxon.

Trend of Na^+^ accumulation in leaves of the model plants treated with NaCl showed a typical saturation-type curve (Figure 8A). Both *Beta* taxa showed higher Na^+^ accumulation potential than that for *C. officinalis* and *M. aquatica*. The accumulation of K^+^ in KCl-treated plants was more pronounced and distinctly more linear than that for Na^+^ (Figure 8B). However, the K^+^ accumulation response was saturable at 100 mmol L^−1^ KCl. The highest K^+^ accumulation was evident for BVC plants. Treatment with NaCl resulted in a significant decrease in K^+^ concentration in the leaves *of M. aquatica* plants at all concentrations and for BVC plants at the highest concentration, but not in BVM and *C. officinalis* plants (Figure 9A). Similarly, Na^+^ concentration significantly decreased due to KCl treatment in all plants besides *C. officinalis* (Figure 9B).

Detailed information on the Na^+^ and K^+^ concentrations in different parts is given in Tables S6–S9. For *Beta* taxa, both senescent and old leaves of NaCl-treated plants had the highest Na^+^ accumulation potential, and more Na^+^ accumulated in petioles in comparison to that in blades. However, this relationship was not pronounced for K^+^ accumulation in KCl-treated *Beta* plants. However, more K^+^ accumulated in the leaf petioles than in the leaf blades of *C. officinalis* plants, and the K^+^ concentration in leaf petioles increased even in NaCl-treated plants (Table S8). Relatively low concentrations of Na^+^ and K^+^ accumulated in the roots of all plant taxa treated with NaCl or KCl, respectively.

**Figure 10:**
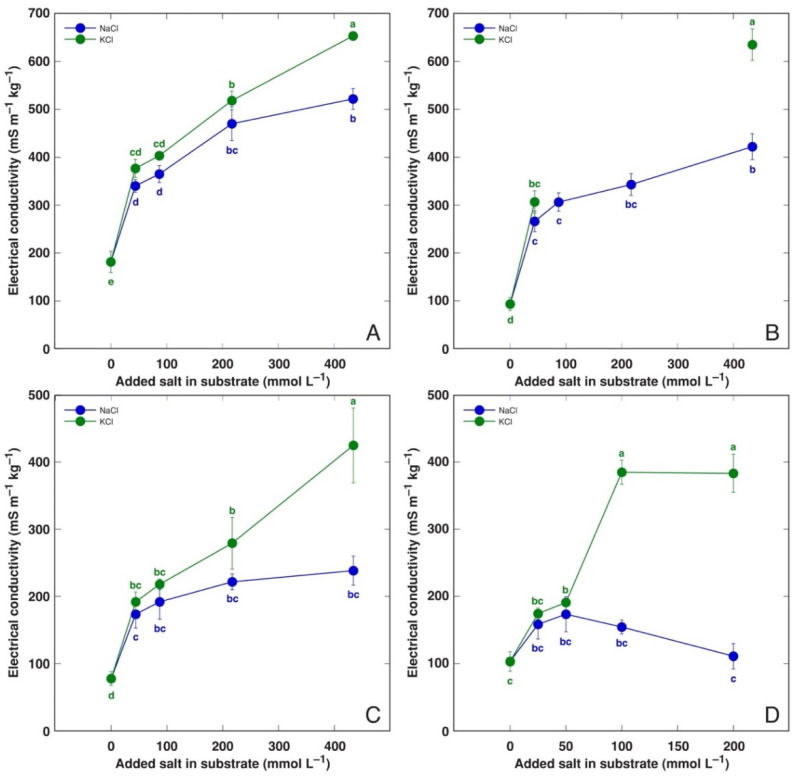
Effect of treatment with different doses of NaCl and KCl on electrical conductivity in leaf extracts of *Beta vulgaris* subsp. *vulgaris* var. *cicla* (old leaf blades; (**A**)), *Beta vulgaris* subsp. *maritima* (old leaf blades; (**B**)), *Cochlearia officinalis* (leaf blades; (**C**)) and *Mentha aquatica* (leaves; (**D**)) plants. Different letters indicate statistically significant (*p* < 0.05) differences between treatments.

Total concentrations of soluble ions, as represented by tissue EC levels, tended to be higher in the leaves of KCl-treated plants in comparison to those in NaCl-treated plants, and this effect was statistically significant at 434 mmol L^−1^ for BVC (Figure 10A), 434 mmol L^−1^ BVM (Figure 10B), 434 mmol L^−1^ *C. officinalis* (Figure 10C) and 100 and 200 mmol L^−1^ for *M. aquatica* (Figure 10D) plants. The dose–response of EC for NaCl-treated plants showed saturation at 217 mmol L^−1^ for both *Beta* taxa and even at 87 mol L^−1^ for *C. officinalis*. The level of EC in the leaves of NaCl-treated *M. aquatica* plants showed some tendency to increase, but the effect was not statistically significant. Detailed information on EC values in different parts is given in Tables S6–S9. In general, EC was higher in the respective parts of equimolar KCl-treated plants in comparison with NaCl-treated plants of all taxa. There was a pronounced gradient of EC from older to younger leaves in NaCl-treated *Beta* plants, especially for BVC, but this was less pronounced in KCl-treated *Beta* plants (Tables S6 and S7).

**Figure 11:**
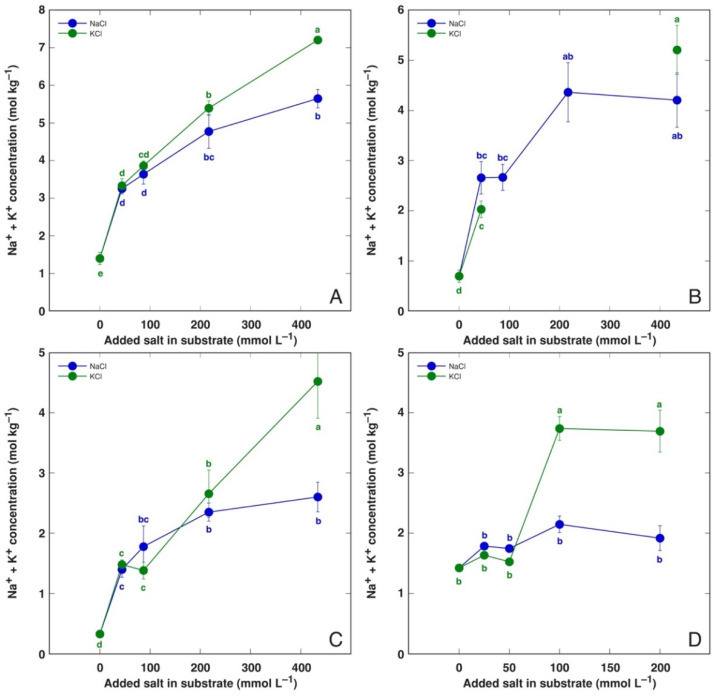
Effect of treatment with different doses of NaCl and KCl on Na^+^ + K^+^ concentration in leaf extracts of *Beta vulgaris* subsp. *vulgaris* var. *cicla* (**A**), *Beta vulgaris* subsp. *maritima* (**B**), *Cochlearia officinalis* (**C**) and *Mentha aquatica* (**D**) plants. Different letters indicate statistically significant (*p* < 0.05) differences between treatments.

The trend of summed Na^+^ + K^+^ concentration with increasing salinity was similar to that established for the EC values (Figure 11). A significant difference between NaCl- and KCl-treated plants was evident at 434 mmol L^−1^ for BVC and *C. officinalis* plants, and at 217 and 434 mmol L^−1^ for *M. aquatica* plants, but not for BVM plants. There was a very tight relationship between the tissue EC value and the summed Na^+^ + K^+^ concentration in all parts of both *B. vulgaris* taxa and *C. officinalis* (Table 2). However, this relationship was less pronounced in *M. aquatica* plants, especially in the stems and roots.

### 3.3. Comparison of NaCl and KCl Effects

The relative effect of NaCl and KCl treatment was evaluated by comparing the maximum total biomass increase at low salinity, the maximum total biomass decrease at high salinity, the maximum increase in H_2_O content in leaf blades as well as the maximum increase in EC values in leaf blades (Table 3). It is evident that plant growth responses, both positive and negative, did not differ between the two cations. No significant differences between the increase in leaf blade H_2_O content were evident for *C. officinalis* and *P. maritima* plants, and only for *M. aquatica* did the K^+^ treatment result in a higher increase in leaf water content. However, the increase in tissue EC value was significantly more pronounced in the case of KCl than for NaCl for all analyzed plant taxa.

## 4. Discussion

Among the model plants used in the present study, both *B. vulgaris* taxa showed the most prominent salinity tolerance, with no negative effect on plant growth even at the highest salinity level (434 mol L^−1^). This is not surprising for *B. vulgaris* subsp. *maritima* as this taxon has been found growing naturally in different salt-affected coastal habitats [19]. However, usually, studies have shown the lower salinity tolerance of different cultivated beet varieties. In a hydroponic culture, the relative growth rate of sea beet was reduced, only not significantly, by 11% at 300 mM salinity, while that of different sugar beet cultivars was reduced by 58–37% [28]. However, in another study, the biomass of *B. vulgaris* subsp. *maritima* plants in gravel hydroponics was reduced by 50% at 375 mol m^−3^ NaCl [22]. In a soil culture, the leaf biomass of leaf beet (*B. vulgaris* var. *cicla*) was already significantly inhibited at 3 g kg^−1^ NaCl in soil, but at 7 g kg^−1^ NaCl, the biomass was inhibited by 75% [47]. In the present study, both *B. vulgaris* taxa were highly tolerant to high salinity caused by NaCl or KCl, showing no negative effect on plant biomass accumulation (Figure 2), but with visible morphological effects (Figures S1 and S2). However, *B. vulgaris* subsp. *maritima* had a tendency to respond more by the increase in biomass at low salinities (Table 3). In general, these findings support the view that the initial salt-tolerance-related characteristics of wild ancestors of beet crops have been preserved during domestication and further breeding process [28].

Both *C. officinalis* and *P. maritima* are coastal-specific plant species and have been classified as typical halophytes [31,40]. While the salinity tolerance of the two species in the conditions of the present study was relatively similar (59–66% and 49–51% biomass decrease at 434 mmol L^−1^ salinity for *C. officinalis* and *P. maritima*, respectively; Table 3), *P. maritima* plants showed a more pronounced biomass increase in the low-salinity treatment (Table 3).

*M. aquatica* plants in the present study showed the highest sensitivity even to low Na^+^ and K^+^ treatment (Figure 2D), and plant shoots almost completely dried out at the highest salinity level (Figure 7). One possibility is that the particular accession of *M. aquatica* does not represent a coastal-specific ecotype, as the general salinity tolerance of the species cannot be expected to be high [34]. However, it cannot be excluded that such a difference from other model plants could be due to the fact that *M. aquatica* plants were cultivated in hydroponics owing to their natural occurrence in a shallow brackish water lagoon. There has not been much of direct comparison between the two systems, but plants in hydroponic conditions are shown to be more sensitive to salinity in comparison to soil cultures, and genotypic variation in cultivars in salt tolerance has been larger in soil in comparison to that in hydroponics, as shown for barley [48]. Therefore, it has been suggested that fundamental differences exist between the two systems of cultivation affecting plant responses to salinity [48].

The ion accumulation potential in the leaves of the studied species was relatively high, reaching 5 mol kg^−1^ Na^+^ for *B. vulgaris* subsp. *vulgaris* var. *cicla*, 4 mol kg^−1^ Na^+^ for B*. vulgaris* subsp. *maritima*, more than 2 mol kg^−1^ Na^+^ for *C. officinalis* and near 2 mol kg^−1^ Na^+^ for *M. aquatica* (Figure 8A). The accumulation potential for K^+^ was higher than that for Na^+^, but mostly only at the highest salinity level (Figure 8B). In previous studies, different Na^+^ accumulation potentials have been reported for these plants. In hydroponics, *C. officinalis* accumulated 5.7 mol kg^−1^ Na^+^ [30]. *B. vugaris* subsp. *vulgaris* var. *cicla* plants accumulated up to 3.5 mol kg^−1^ Na^+^ in alkaline conditions [25], but only 0.7 mol kg^−1^ Na^+^ was found for *B. vulgaris* subsp. *maritima* [22]. It is evident that very high variability exists between various studies with the same plant species, mainly due to differences in experimental conditions, sampling procedures, etc. However, genotype-specific effects cannot be excluded.

The ability of most plants to accumulate supraoptimal concentrations of K^+^ in tissues is a well-known fact, and it has been associated with predominant vacuolar sequestration, acting as a reserve pool of K^+^ [49]. It is evident that Na^+^ sequestration in vacuoles of salt-tolerant accumulating plant species largely resembles this response. Indeed, the existence of shared transport systems within the plant for the two cations has been indeed shown [50], including these located in the tonoplast [51]. Interestingly, for all model plants in the present study, the K^+^ accumulation ability was consequently more pronounced than that of Na^+^ when the same plant was cultivated at the same molar concentration of KCl or NaCl, respectively (Figure 8), and this resulted in higher values of tissue EC (Figure 10, Table 2). However, the difference was only significant at high salinity.

An increase in tissue water content in plants at elevated substrate salinity is a part of an adaptive mechanism of induced succulence, as indicated already by early studies [52]. Salt dilution by increased water content is among most important adaptive mechanisms of salt-accumulating halophyte species [53,54]. In the present study, the increase in leaf water content by increasing substrate salinity was the most pronounced in both *B. vulgaris* taxa at all NaCl and KCl concentrations (Figure 4 and Figure 5). Similarly, both leaf thickness and their succulence significantly increased in *B. vulgaris* subsp. *maritima* and various sugar beet cultivars with increasing salinity in hydroponic conditions [28]. A decrease in leaf water content by 434 mmol L^−1^ KCl treatment in *C. officinalis* plants (Figure 6) and by relatively high salinity in *M. aquatica* plants (Figure 7) most likely reflected the induction of senescence as a result of prolonged salinity, as indicated by other studies [55].

One of possible reasons for the negative effect of Na^+^ on plant growth (Na^+^ “toxicity”) is mentioned to be a reduction in the K^+^ concentration in tissues. Significant growth reduction in *P. maritima* plants at a NaCl concentration above 200 mM had been suggested to be a result of a decrease in K^+^ uptake [42]. However, Na^+^ and K^+^ clearly had similar effects on the growth of *P. maritima* (Figure 1B) and other model plants (Figure 2) of the present study, as summarized in Table 2. However, the increase in the total electrolytic activity in plant tissues was more pronounced for KCl-treated plants (Figure 8, Table 3).

Previously, only obligate halophyte species, such as *Atriplex nummularia, Atriplex halimus* and *Sesuvium portulacastrum*, were shown to have similar morphological responses to NaCl and KCl treatment [11,12,13]. Recently, it was established that NaCl and KCl had similar effects on the growth of three accessions of facultative recretohalophyte *Armeria maritima* from isolated micropopulations located in sandy soil habitats [56]. However, both cations differentially affected osmotic adjustment in leaf tissues: while the osmotic value increased in both treatments in a concentration-dependent manner, only plants treated with NaCl showed a significant increase in the non-ionic osmotic value. Consequently, together with the data of the present study, these results suggest that taxonomically different plant species possessing rather variable levels of salinity tolerance respond equally to NaCl and KCl treatment at the morphological level but may have differences in protection mechanisms of the internal environment.

The results of the present study clearly showed that, for a number of putatively salt-tolerant, taxonomically different plant taxa, NaCl and KCl treatment had similar genotype-specific and concentration-dependent effects on plant growth and development. This can be explained by two non-mutually exclusive possibilities: (i) the level of “toxicity” of Na^+^ and K^+^ is identical and not specific and (ii) Cl^−^ is the element resulting in “toxicity”. These possibilities will be discussed in detail further.

A well-known fact from plant mineral nutrition experiments and fertilization practice shows that a too-high (supraoptimal) concentration of minerals significantly reduces plant growth and causes other signs of toxicity [57]. There is a reason to believe that plant sensitivity vs. tolerance to a high soil electrolyte level is a species-specific trait, allowing us to define “eletrolytophytic species” as the ones with a high general tolerance to salinity and an ability to accumulate high electrolyte concentrations in photosynthetically active tissues [16]. So far, comparative experimental evidence for plant genetic differences in degree of electrolyte tolerance and/or accumulation is rather limited. Electrolyte accumulation potential in plant leaves significantly varied within 102 species from salt-affected coastal habitats, but the particular level of tissue EC did not show a general relation with the putative salinity tolerance of the species [16]. It seems that the major electrolytically active ions were Na^+^, K^+^ and Cl^−^, as evident by the relatively tight relationship between the summed Na^+^ plus K^+^ concentration (which in fact also included a Cl^−^ component) and tissue EC, especially in leaves (Table 2). Therefore, a characteristic decrease in electrolyte concentration from older to younger leaves possibly reflects a lower degree of the vacuolation of mesophyll cells in younger tissues [58]. However, this feature was not evident for the accumulation of K^+^ in KCl-treated plants, pointing to other possible sites of surplus K^+^ accumulation besides vacuoles. Preferential Na^+^ accumulation in older leaves of accumulating halophyte species has also been noted in a number of previous studies [56,58]. This type of accumulation is also characteristic for heavy metals, which is especially pronounced in plants with a rosette type of growth, allowing for induced senescence and the gradual replacement of older metal-accumulating leaves [59].

The chaotropic effect of ions as a mechanism of salt toxicity cannot be ruled out. This idea has been tested with extremophile microorganisms, and several species of fungi were shown to be chaotolerant [60]. While NaCl is sometimes considered a chaotropic salt, Na^+^ itself is kosmotropic (being strongly hydrated), but both Cl^−^ and K^+^ are chaotropic (being weakly hydrated), as indicated by the respective Jones–Doyle viscosity coefficients [61]. Therefore, theoretically, KCl might have a stronger chaotropic effect in comparison to that of NaCl. However, due to physiological mechanisms of ion compartmentation, the accumulation of K^+^ at concentrations high enough to cause adverse chaotropic effects is highly unlikely. This inevitably leads back to the idea that it is Cl^−^ that is the potentially toxic element in the salinity of chloride salts.

Cl^−^ itself is considered to represent an essential plant nutrient [62], but it is still a matter of debate as to whether it represents a micronutrient or beneficial macronutrient [63]. The possible negative effect of Cl^−^ in NaCl-treated plants usually has been neglected in the majority of studies, but see Martin, Koebner 1995 [64]. Only recently has a deeper interest in Cl^−^ effects been renewed [65,66,67,68,69]. On a positive side, in addition to the established role of Cl^−^ in photosynthesis [70], the importance of osmotic adjustment, the control of turgor and water balance has been suggested [71]. Additional functions of Cl^−^ can be proposed for halophytic species [67]. On a negative side, competition of Cl^−^ with anionic forms of nutrients, nitrate and phosphate, can significantly disturb plant mineral nutrition, leading to reduced growth [66]. In typical glycophytes species, it was shown that Na^+^ and Cl^−^ synergistically contribute to salt toxicity [64]. Sugar beet is listed among the crop plants with the highest Cl^−^ tolerance level, with no negative effects seen even at a 50.8 g kg^−1^ tissue concentration of Cl^−^ [62]. Both Swiss chard and table beet plants had a maximum growth increase at 80 mol m^−3^ NaCl, being 146 and 128%, respectively [72]. However, a change in the Na^+^ vs. K^+^ external ratio at identical salinity had no effect on the growth of the two plants [73]. These findings are fully comparable with the results of the present study, showing the extremely high tolerance of the two *B. vulgaris* taxa to Na^+^ and K^+^ chloride salinity, with no large differences in the effect of the two cations. However, at equimolar concentrations, NO_3_^−^ uptake was significantly enhanced in NaCl-treated *B. vulgaris* var. *cicla* plants but not in the KCl-treated ones [74].

Salt stress sensing and the hormonal regulation of salinity tolerance responses in plants are still underexplored directions in the biology of plant adaptations to environmental heterogeneity. To dissect the general osmotic, electrolytic or ion-specific effects of salinity responses, more attention needs to be forwarded to comparative studies involving plant taxa with variable salinity tolerance affected by different combinations of salts. The use of these model systems together with approaches of transcriptomics and ionomics will allow us to better understand plant adaptation mechanisms to different types of salinity.

## 5. Conclusions

Taxonomically different plant species showed similar growth responses to Na^+^ and K^+^ salinity in a form of chloride salts. While the morphological responses of *B. vulgaris* subsp. *vulgaris* var. *cicla*, *B. vulgaris* subsp. *maritima* and *P. maritima* plants to NaCl and KCl were rather similar, *C. officinalis* plants tended to perform worse when treated with KCl, but the opposite was evident for *M. aquatica*. Based on the results of the present study, it seems that, at least for the model plants used, both positive and negative effects of Na^+^ and K^+^ on plant growth were due to the unspecific ionic effects of monovalent cations or/and specific effects of Cl^−^.

## Figures and Tables

**Table 1:** Studied plants, their origin and experimental detail.

Code	Model Plant	Propagation Material (Source)	Cultivation System	Treatments	Analyzed Parameters
BVC	*Beta vulgaris* subsp. *vulgaris* L. var. *cicla* cv. ‘Magenta Sunset’	Seeds (Sėklos, Vilnius, Lithuania)	Garden soil/quartz sand 3:1 (*v*/*v*)	NaCl (44, 87, 217, 434 mmol L^−1^). KCl (44, 87, 217, 434 mmol L^−1^)	Dry biomass, H_2_O, Na, K, EC
BVM	*Beta vulgaris* subsp. *maritima* (L.) Arcang.	Seeds (Agroforestry Research Trust, Dartington, Devon, UK)	Garden soil/quartz sand 3:1 (*v*/*v*)	NaCl (44, 87, 217, 434 mmol L^−1^). KCl (44, 434 mmol L^−1^)	Dry biomass, H_2_O, Na, K, EC
CO	*Cochlearia officinalis* L.	Seeds (Jelitto Staudensamen, Schwarmstedt, Germany)	Garden soil/quartz sand 5:1 (*v*/*v*)	NaCl (44, 87, 217, 434 mmol L^−1^). KCl (44, 87, 217, 434 mmol L^−1^)	Dry biomass, H_2_O, Na, K, EC
MA	*Mentha aquatica* L.	Stem explants (seawater-affected sandy beach, Ainaži, Latvia)	Hydroponics, Kristalon Red + Calcinit (0.5 g L^−1^)	NaCl (25, 50, 100, 200 mmol L^−1^). KCl (25, 50, 100, 200 mmol L^−1^)	Dry biomass, H_2_O, Na, K, EC
PM	*Plantago maritima* L.	Seeds (seawater-affected gravel beach, Ohesaare, island of Saaremaa, Estonia)	Garden soil/quartz sand 3:1 (*v*/*v*)	NaCl (22, 44, 87, 217, 434 mmol L^−1^). KCl (22, 44, 87, 217, 434 mmol L^−1^)	Dry biomass, H_2_O

EC, electrical conductivity.

**Table 2:** Relationship between tissue electrical conductivity (EC) and summed concentration of Na^+^ + K^+^ in different parts of the model plants.

Model Plant	Plant Part	*R* ^2^	*r*
BVC	Leaf petioles	0.978	0.989
	Leaf blades	0.984	0.992
	Roots	0.970	0.985
BVM	Leaf petioles	0.873	0.934
	Leaf blades	0.860	0.927
	Roots	0.863	0.929
CO	Leaf petioles	0.995	0.997
	Leaf blades	0.982	0.991
	Roots	0.970	0.985
MA	Stems	0.548	0.741
	Leaves	0.822	0.907
	Roots	0.625	0.790

BVC, Beta vulgaris subsp. maritima; BVM, Beta vulgaris subsp. vulgaris var. cicla; CO, Cochlearia officinalis; MA, Mentha aquatica.

**Table 3:** Comparison of the main relative effects of NaCl and KCl in the model plants. EC, electrical conductivity.

Parameter	Model Plant	Effect of NaCl	Effect of KCl	Na^+^ vs. K^+^
Maximum dry mass increase (%)	BVC	37	20	n.s.
	BVM	47 *	29	n.s.
	CO	13	7	n.s.
	MA	0	0	n.s.
	PM	29	46 *	n.s.
Maximum dry mass decrease (%)	BVC	16	15	n.s.
	BVM	18	15	n.s.
	CO	59 *	66 *	n.s.
	MA	65 *	65 *	n.s.
	PM	51 *	49 *	n.s.
Maximum increase in H_2_O in leaf blades (%)	BVC	96 *	78 *	n.s.
	BVM	91 *	(54 *)	-
	CO	45 *	18	n.s.
	MA	43 *	97 *	s.
	PM	60 *	56 *	n.s.
Maximum increase in EC in leaf blades (%)	BVC	187 *	260 *	s.
	BVM	351 *	579 *	s.
	CO	207 *	447 *	s.
	MA	68	272 *	s.

Significant differences from the respective control are indicated by * (*p* < 0.05). n.s., not significant (*p* ≥ 0.05); s., significant, *p* < 0.05). For BVM plants, K^+^ concentration resulting in maximum increase in H_2_O content was not used. BVC, *Beta vulgaris* subsp. *maritima*; BVM, *Beta vulgaris* subsp. *vulgaris* var. *cicla*; CO, *Cochlearia officinalis*; MA, *Mentha aquatica*.

## Data Availability

All data reported here are available from the authors upon request.

## Supplementary Materials

The following are available online at https://www.mdpi.com/article/10.3390/life12101577/s1, Figure S1: Typical *Plantago maritima* plants 6 weeks after the final treatment with NaCl (A) and KCl (B), Figure S2: Typical *Beta vulgaris* subsp. *vulgaris* var. *cicla* plants 4 weeks after the final treatment with NaCl (A) and KCl (B), Figure S3: Typical *Beta vulgaris* subsp. *maritima* plants 5 weeks after the final treatment with NaCl and KCl, Figure S4: Typical *Cochlearia officinalis* plants 3 weeks after the final treatment with NaCl (A) and KCl (B), Table S1: Effect of NaCl and KCl treatment on morphological parameters of *Plantago maritima* plants, Table S2: Effect of NaCl and KCl treatment on morphological parameters of *Beta vulgaris* subsp. *vulgaris* var. *cicla*, Table S3: Effect of NaCl and KCl treatment on morphological parameters of *Beta vulgaris* subsp. *maritima* plants, Table S4: Effect of NaCl and KCl treatment on morphological parameters of *Cochlearia officinalis* plants, Table S5: Effect of NaCl and KCl treatment on morphological parameters of *Mentha aquatica* plants, Table S6: Effect of NaCl and KCl treatment on Na^+^ and K^+^ concentration and electrical conductivity (EC) in different parts of *Beta vulgaris* subsp. *vulgaris* var. *cicla,* Table S7: Effect of NaCl and KCl treatment on Na^+^ and K^+^ concentration and electrical conductivity (EC) in different parts of *Beta vulgaris* subsp. *maritima,* Table S8: Effect of NaCl and KCl treatment on Na^+^ and K^+^ concentration and electrical conductivity (EC) in different parts of *Cochlearia officinalis,* Table S9: Effect of NaCl and KCl treatment on Na^+^ and K^+^ concentration and electrical conductivity (EC) in different parts of *Mentha aquatica.*

## References

[1] van Zelm E., Zhang Y., Testerink C. (2020). Salt tolerance mechanisms of plants. Annu. Rev. Plant Biol..

[2] Fang S., Hou X., Liang X. (2021). Response mechanisms of plants under saline-alkali stress. Front. Plant Sci..

[3] Hameed A., Ahmed M.Z., Hussain T., Aziz I., Ahmad N., Gul B., Nielsen B.L. (2021). Effects of salinity stress on chloroplast structure and function. Cells.

[4] Rahman M.M., Mostofa M.G., Keya S.S., Siddiqui M.N., Ansary M.M., Das A.K., Rahman M.A., Tran L.S.-P. (2022). Adaptive mechanisms of halophytes and their potential in improving salinity tolerance in plants. Int. J. Mol. Sci..

[5] Soltabayeva A., Ongaltay A., Omondi J.O., Srivastava S. (2021). Morphological, physiological and molecular markers for salt-stressed plants. Plants.

[6] Valenzuela F.J., Reineke D., Leventini D., Chen C.C.L., Barrett-Lennard E.G., Colmer T.D., Dodd I.C., Shabala S., Brown P., Bazihizina N. (2022). Plant responses to heterogeneous salinity: Agronomic relevance and research priorities. Ann. Bot..

[7] Ondrasek G., Rengel Z. (2021). Environmental salinization processes: Detection, implications & solutions. Sci. Total Environ..

[8] Maathuis F.J.M., Amtmann A. (1999). K^+^ nutrition and Na^+^ toxicity: The basis of cellular K^+^/Na^+^ ratios. Ann. Bot..

[9] Kronzucker H.J., Coskun D., Schulze L.M., Wong J.R., Britto D.T. (2013). Sodium as nutrient and toxicant. Plant Soil.

[10] Martínez-Ballesta M.C., Martínez V., Carvajal M. (2004). Osmotic adjustment, water relations and gas exchange in pepper plants grown under NaCl and KCl. Environ. Exp. Bot..

[11] Ramos J., López M.J., Benlloch M. (2004). Effect of NaCl and KCl salts on the growth and solute accumulation of the halophyte *Atriplex nummularia*. Plant Soil.

[12] Wang D., Wang H., Han B., Wang B., Guo A., Zheng D., Liu C., Chang L., Peng M., Wang X. (2012). Sodium instead of potassium and chloride is an important macronutrient to improve leaf succulence and shoot development for halophyte *Sesuvium portulacastrum*. Plant Physiol. Biochem..

[13] Belkheiri O., Mulas M. (2013). The effects of salt stress on growth, water relations and ion accumulation in two halophyte *Atriplex* species. Environ. Exp. Bot..

[14] Zhao C., Zhang H., Song C., Zhu J.-K., Shabala S. (2020). Mechanisms of plant responses and adaptation to soil salinity. Innovation.

[15] Surówka E., Hura T., Grigore M.-N. (2021). Osmoprotectants and nonenzymatic antioxidants in halophytes. Handbook of Halophytes.

[16] Ievinsh G., Ieviņa S., Andersone-Ozola U., Samsone I. (2021). Leaf sodium, potassium and electrolyte accumulation capacity of plant species from salt-affected coastal habitats of the Baltic Sea: Towards a definition of Na hyperaccumulation. Flora.

[17] Leys M., Petit E.J., El-Bahloul J., Liso C., Fournet S., Arnaud J.-F. (2014). Spatial genetic structure in *Beta vulgaris* subsp. *maritima* and *Beta macrocarpa* reveals the effect of contrasting mating system, influence of marine currents, and footprints of postglacial recolonization routes. Ecol. Evol..

[18] Andrello M., Henry K., Devaux P., Desprez B., Manel S. (2015). Taxonomic, spatial and adaptive genetic variation of *Beta* section *Beta*. Theor. Appl. Genet..

[19] Frese L., Ford-Lloyd B., Biancardi E., Panella L.W., McGrath J.M. (2020). Range of distribution. Beta maritima, the Origin of Beets.

[20] Frese L., Ford-Lloyd B., Biancardi E., Panella L.W., McGrath J.M. (2020). Taxonomy, phylogeny, and the genepool. Beta maritima, the Origin of Beets.

[21] Bor M., Özdemir F., Türkan I. (2003). The effect of salt stress on lipid peroxidation and antioxidants in leaves of sugar beet *Beta vulgaris* L. and wild beet *Beta maritima* L. Plant Sci..

[22] Koyro H.-W., Daoud S., Harrouni C., Huchzermeyer B. (2006). Strategies of a potential cash crop halophyte (*Beta vulgaris* ssp. maritima) to avoid salt injury. Trop. Ecol..

[23] Ribeiro I.C., Pinheiro C., Ribeiro C.M., Veloso M., Simões-Costa M.C., Evaristo I., Paulo O.S., Ricardo C.P. (2016). Genetic diversity and physiological performance of Portuguese wild beet (*Beta vulgaris* spp. *maritima*) from three contrasting habitats. Front. Plant Sci..

[24] Skorupa M., Gołȩbiewski M., Kurnik K., Niedojadło J., Kȩsy J., Klamowski K., Wójcik K., Treder W., Tretyn A., Tyburski J. (2019). Salt stress vs. salt shock—The case of sugar beet and its halophytic ancestor. BMC Plant Biol..

[25] Liu L., Ueda A., Saneoka H. (2013). Physiological responses of white Swiss chard (*Beta vulgaris* L. subsp. *cicla*) to saline and alkaline stress. Austr. J. Crop Sci..

[26] Kaburagi E., Morikawa Y., Yamada M., Fujiyama H. (2014). Sodium enhances nitrate uptake in Swiss chard (*Beta vulgaris* var. *cicla* L.). Soil Sci. Plant Nutr..

[27] Puccinelli M., Carmassi G., Botrini L., Bindi A., Rossi L., Fierro-Sañudo J.F., Pardossi A., Incrocci L. (2022). Growth and mineral relations of *Beta vulgaris* var. *cicla* and *Beta vulgaris* ssp. *maritima* cultivated hydroponically with diluyed seawater and low nitrogen level in the nutrient solution. Horticulturae.

[28] Rozema J., Cornelisse D., Zhang Y., Li H., Bruning B., Katschnig D., Broekman R., Ji B., van Bodegom P. (2014). Comparing salt tolerance of beet cultivars and their halophytic ancestor: Consequences of domestication and breeding programmes. AoB Plants.

[29] Nawaz I., Iqbal M., Bliek M., Schat H. (2017). Salt and heavy metal tolerance and expression levels of candidate tolerance genes among four extremophile *Cochlearia* species with contrasting habitat preferences. Sci. Total Environ..

[30] de Vos A.C., Broekman R., de Almeida Guerra C.C., van Rijsselberghe M., Rozema J. (2013). Developing and testing new halophyte crops: A case study of salt tolerance of two species of the Brassicaceae, *Diplotaxis tenuifolia* and *Cochlearia officinalis*. Environ. Exp. Bot..

[31] Nordal I., Eriksen A.B., Laane M.M., Solberg Y. (1986). Biogeographic and biosystematic studies in the genus *Cochlearia* in Northern Scandinavia. Acta Univ. Upsaliensis. Symb. Bot. Ups..

[32] Janssen J.A.M., Rodwell J.S. (2016). European Red List of Habitats: Part 2. Terrestrial and Freshwater Habitats.

[33] Puijalon S., Bouma T.J., van Groenendael J., Bornette G. (2008). Clonal plasticity of aquatic plant species submitted to mechanical stress: Escape versus resistance strategy. Ann. Bot..

[34] Haddadi B.S., Hassanpour H., Niknam V. (2016). Effect of salinity and waterlogging on growth, anatomical and antioxidative responses in *Mentha aquatica* L. Acta Physiol. Plant..

[35] Sniedze-Kretalova R., Auniņš A. (2013). 6430. Hydrophilous tall herb fringe communities of plains and the montane to alpine levels. European Union Protected Habitats in Latvia. Interpretation Manual.

[36] Rūsiņa S., Auniņš A. (2013). 6430. Water courses of plain to montane levels with the Ranunculion fluitantis and Callitricho-Batrachion vegetation. European Union Protected Habitats in Latvia. Interpretation Manual.

[37] Kalniņš M., Andersone-Ozola U., Gudrā D., Sieriņa A., Fridmanis D., Ievinsh G., Muter O. (2022). Effect of bioaugumentation on the growth and rhizosphere microbiome assembly of hydroponic cultures of *Mentha aquatica*. Ecol. Genet. Genom..

[38] Erdei L., Kuiper P.J.C. (1979). The effect of salinity on growth, cation content, Na^+^ uptake and translocation in salt-sensitive and salt-tolerant *Plantago* species. Physiol. Plant..

[39] Tánczos O.G., Erdei L., Snijder J. (1981). Uptake and translocation of sodium in salt-sensitive and salt-tolerant *Plantago* species. Plant Soil.

[40] Königshofer H. (1983). Changes in ion composition and hexitol content of different *Plantago* species under the influence of salt stress. Plant Soil.

[41] Blom C.W.P.M., Kuiper P.C.J., Bos M. (1992). Phenotypic plasticity in *Plantago maritima*. Plantago: A Multidisciplinary Study.

[42] Sleimi N., Guerfali S., Bankaji I. (2015). Biochemical indicators of salt stress in *Plantago maritima*: Implications for environmental stress assessment. Ecol. Indic..

[43] Rubinigg M., Posthumus F., Ferschke M., Elzenga J.T.M., Stulen I. (2003). Effects of NaCl salinity on ^15^N-nitrate fluxes and specific root length in the halophyte *Plantago maritima* L. Plant Soil.

[44] Jerling L. (1984). The impact of some environmental factors on the establishment of *Plantago maritima* seedlings and juveniles along a distributional gradient. Holarctic Ecol..

[45] Jerling L. (1985). Population dynamics of *Plantago maritima* along a distributional gradient on a Baltic seashore meadow. Vegetatio.

[46] Jerling L., Liljelund L.-E. (1984). Dynamics of *Plantago maritima* along a distributional gradient: A demographic study. Holarctic Ecol..

[47] He H., Zhou H., Lü H., Liang B. (2022). Growth, morphological and physiological adaptability of leaf beat (*Beta vulgaris* var. *cicla*) to salt stress: A soil culture experiment. Agronomy.

[48] Tavakkoli E., Fatehi F., Rengasamy P., McDonald G.K. (2012). A comparison of hydroponic and soil-based screening methods to identify salt tolerance in the field in barley. J. Exp. Bot..

[49] Walker D.J., Leigh R.A., Miller A.J. (1996). Potassium homeostasis in vacuolated plant cells. Proc. Natl. Acad. Sci. USA.

[50] Rodríguez-Navarro A., Rubio F. (2006). High-affinity potassium and sodium transport systems in plants. J. Exp. Bot..

[51] Adams E., Shin R. (2014). Transport, signaling, and homeostasis of potassium and sodium in plants. J. Integr. Plant Biol..

[52] Jennings D.H. (1968). Halophytes, succulence and sodium in plants–a unified theory. New Phytol..

[53] Song J., Wang B. (2015). Using euhalophytes to understand salt tolerance and to develop saline agriculture: *Suaeda salsa* as a promising model. Ann. Bot..

[54] Yuan F., Xu X., Leng B., Wang B. (2019). Beneficial effects of salinity on halophyte growth: Morphology, cells, and genes. Open Life Sci..

[55] Allu A.D., Soja A.M., Wu A., Szymanski J., Balazadeh S. (2014). Salt stress and senescence: Identification of cross-talk regulatory components. J. Exp. Bot..

[56] Purmale L., Jēkabsone A., Andersone-Ozola U., Ievinsh G. (2022). Salinity tolerance, ion accumulation potential and osmotic adjustment in vitro and in planta of different *Armeria maritima* accessions from a dry coastal meadow. Plants.

[57] Schulze E.D., Beck E., Buchmann N., Clemens S., Müller-Hohenstein K., Scherer-Lorenzen M. (2019). Adverse soil mineral availability. Plant Ecology.

[58] Bonales-Alatorre E., Shabala S., Chen Z.-H., Pottosin I. (2013). Reduced tonoplast fast-activating and slow-activating channel activity is essential for conferring salinity tolerance in a facultative halophyte, quinoa. Plant Physiol..

[59] Szarek-Łukaszewska G., Słysz A., Wierzbicka M. (2004). Response of *Armeria maritima* (Mill.) Willd. to Cd, Zn and Pb. Acta Biol. Cracov. Ser. Bot..

[60] Zajc J., Džeroski S., Kocev D., Oren A., Sonjak S., Tkavc R., Gunde-Cimerman N. (2014). Chaophilic or chaotolerant fungi: A new category of extremophiles?. Front. Microbiol..

[61] Collins K.D. (2012). Why continuum electrostatic theories cannot explain biological structure, polyelectrolytes or ionic strength effects in ion–protein interactions. Biophys. Chem..

[62] Xu G., Magen H., Tarchitzky J., Kafkafi U. (2000). Advances in chloride nutrition of plants. Advances in Agronomy.

[63] Colmenero-Flores J.M., Franco-Navarro J.D., Cuberto-Font P., Peinado-Torrubia P., Rosales M.A. (2019). Chloride as a beneficial macronutrient in higher plants: New roles and regulation. Int. J. Mol. Sci..

[64] Martin P.K., Koebner R.M.D. (1995). Sodium and chloride ions contribute synergistically to salt toxicity in wheat. Biol. Plant..

[65] Raven J.A. (2017). Chloride: Essential micronutrient and multifunctional beneficial ion. J. Exp. Bot..

[66] Geilfus C.M. (2018). Chloride: From nutrient to toxicant. Plant Cell Physiol..

[67] Bazihizina N., Colmer T.D., Cuin T.A., Mancuso S., Shabala S. (2019). Friend or foe? Chloride patterning in halophytes. Trends Plant Sci..

[68] Flowers T.J., Glenn E.P., Volkov V. (2019). Could vesicular transport of Na^+^ and Cl^−^ be a feature of salt tolerance in halophytes?. Ann. Bot..

[69] Le L.T.T., Kotula L., Siddique K.H.M., Colmer T.D. (2021). Na^+^ and/or Cl^–^ toxicities determine salt sensitivity in soybean (*Glycine max* (L.) Merr.), mungbean (*Vigna radiata* (L.) R. Wilczek), cowpea (*Vigna unguiculata* (L.) Walp.) and common bean (*Phaseolus vulgaris* L.). Int. J. Mol. Sci..

[70] Kawakami K., Umena Y., Kamiya N., Shen J.R. (2009). Location of chloride and its possible functions in oxygen-evolving photosystem II revealed by X-ray crystallography. Proc. Natl. Acad. Sci. USA.

[71] Franco-Navarro J.D., Brumos J., Rosales M.A., Cubero-Font P., Talon M., Colmenero-Flores J.M. (2016). Chloride regulates leaf cell size and water relations in tobacco plants. J. Exp. Bot..

[72] Yamada M., Kuroda C., Fujiyama H. (2016). Growth promotion by sodium in amaranthaceous plants. J. Plant Nutr..

[73] Yamada M., Kuroda C., Fujiyama H. (2016). Function of sodium and potassium in growth of sodium-lowing Amaranthaceae species. Soil Sci. Plant Nutr..

[74] Kaburagi E., Yamada M., Fujiyama H. (2015). Sodium, but not potassium, enhances root to leaf nitrate translocation in Swicc chard (*Beta vulgaris* var. *cicla* L.). Environ. Exp. Bot..

