# Supplementary material for: Similar Responses of Relatively Salt-Tolerant Plants to Na and K during Chloride Salinity: Comparison of Growth, Water Content and Ion Accumulation
Source: Life (Basel). 2022 Oct 11;12(10):1577. doi: 10.3390/life12101577 (PMC9605674; doi:10.3390/life12101577)
Supplement: Supplementary file 1 [file life-12-01577-s001.zip › life-1966976-supplementary.pdf]

Supplement

Similar responses to Na and K during chloride salinity: comparison of growth, water content and ion accumulation of several model plants with relatively high salt tolerance

Figure S1. Typical *Plantago maritima* plants 6 weeks after the final treatment with NaCl (A) and KCl (B).

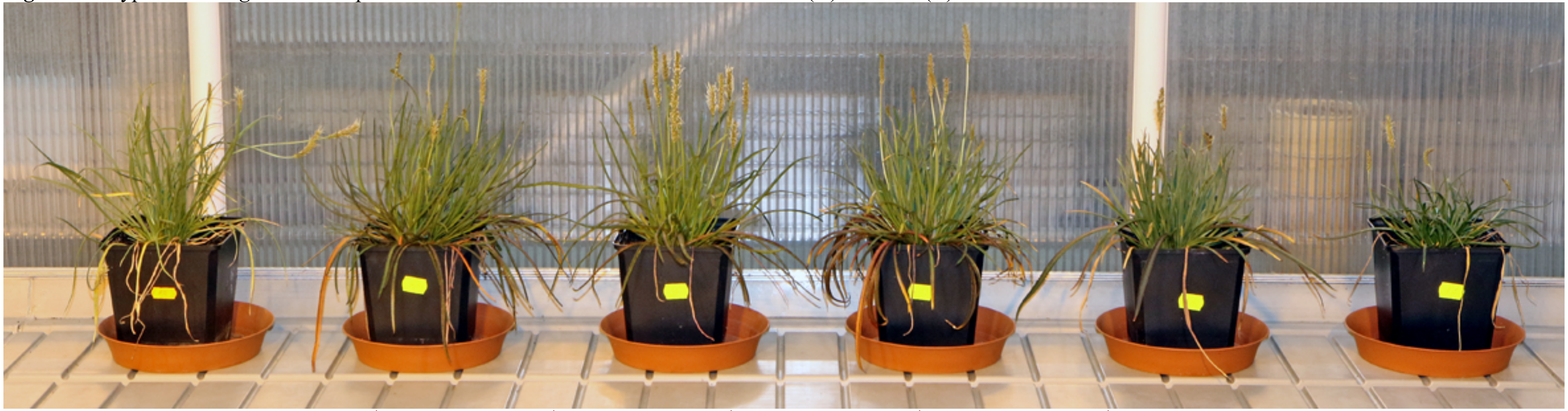

A, from left to right: control, 22 mmol L^{-1} NaCl, 44 mmol L^{-1} NaCl, 88 mmol L^{-1} NaCl, 217 mmol L^{-1} NaCl, 434 mmol L^{-1} NaCl.

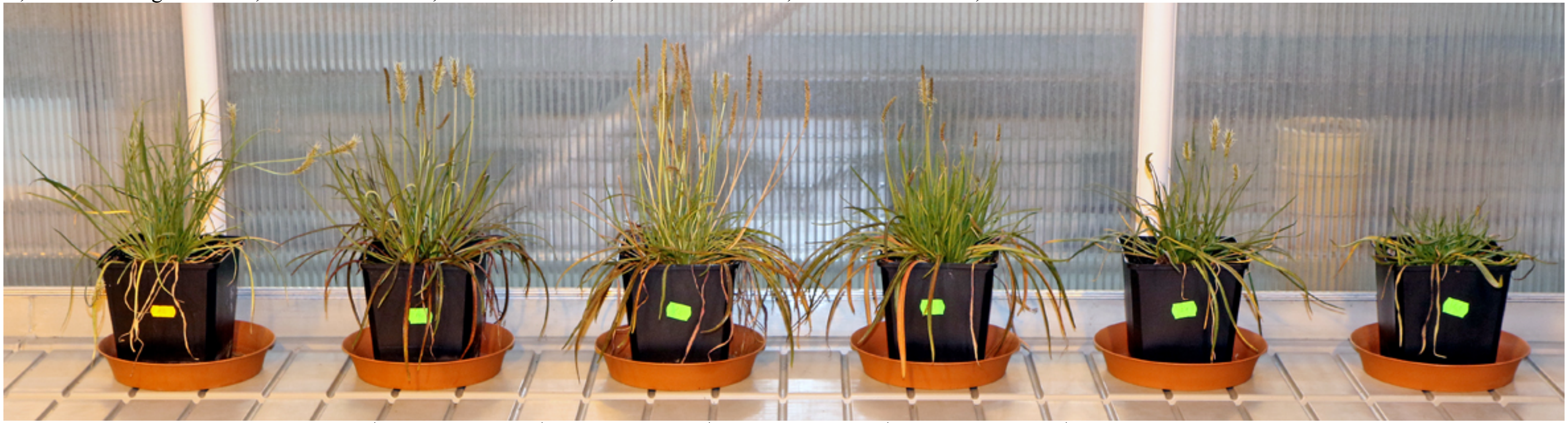

B, from left to right: control, 22 mmol L^{-1} KCl, 44 mmol L^{-1} KCl, 88 mmol L^{-1} KCl, 217 mmol L^{-1} KCl, 434 mmol L^{-1} KCl.

Figure S2. Typical *Beta vulgaris* subsp. *vulgaris* var. *cicla* plants 4 weeks after the final treatment with NaCl (A) and KCl (B).

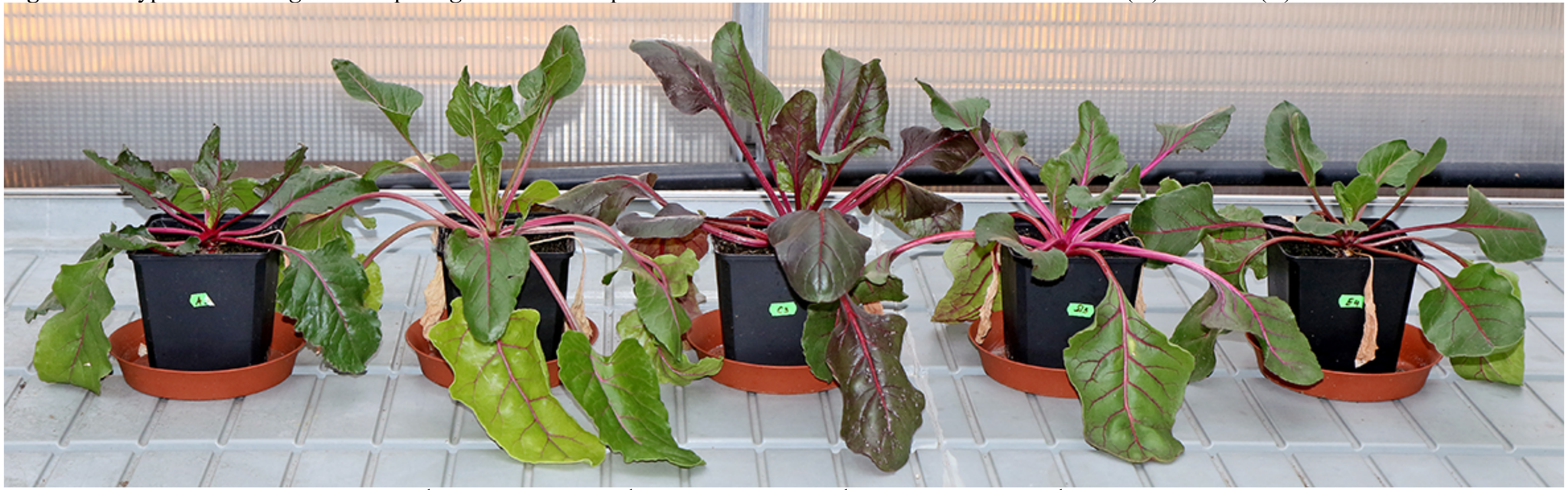

A, from left to right: control, 44 mmol L^{-1} NaCl, 88 mmol L^{-1} NaCl, 217 mmol L^{-1} NaCl, 434 mmol L^{-1} NaCl.

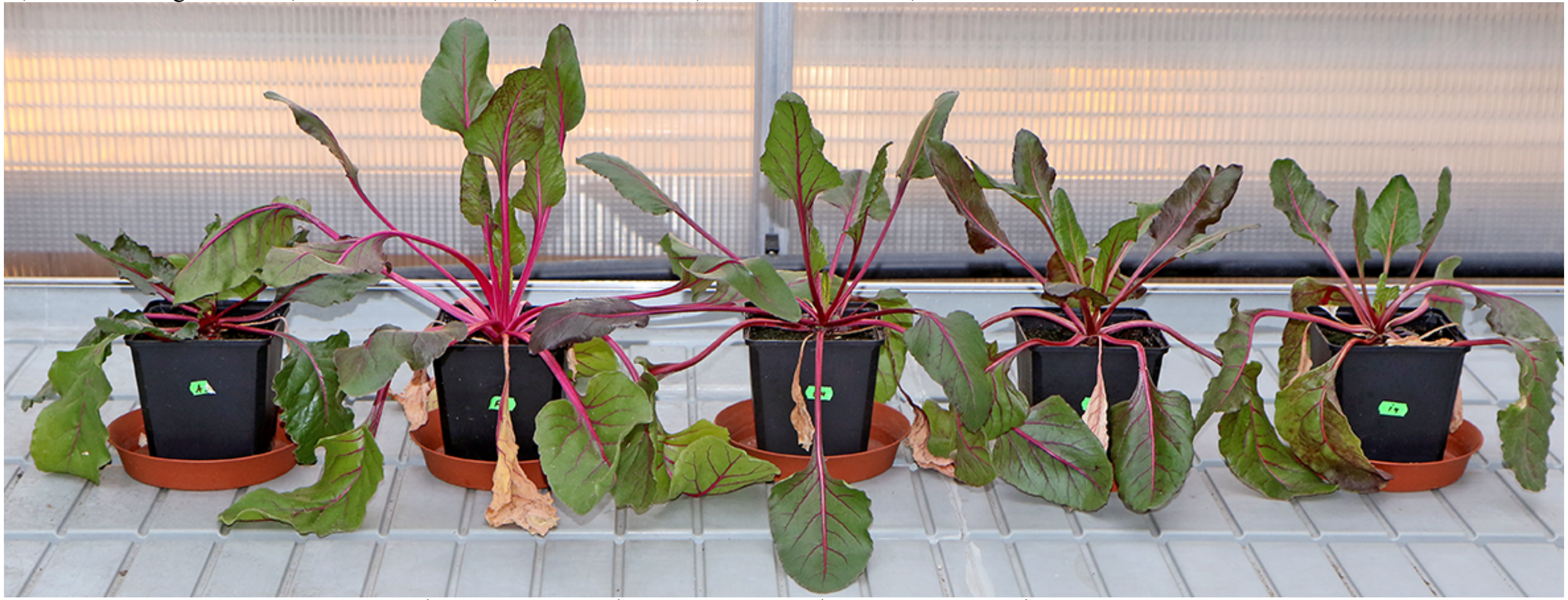

B, from left to right: control, 44 mmol L^{-1} KCl, 88 mmol L^{-1} KCl, 217 mmol L^{-1} KCl, 434 mmol L^{-1} KCl.

Figure S3. Typical *Beta vulgaris* subsp. *maritima* plants 5 weeks after the final treatment with NaCl and KCl.

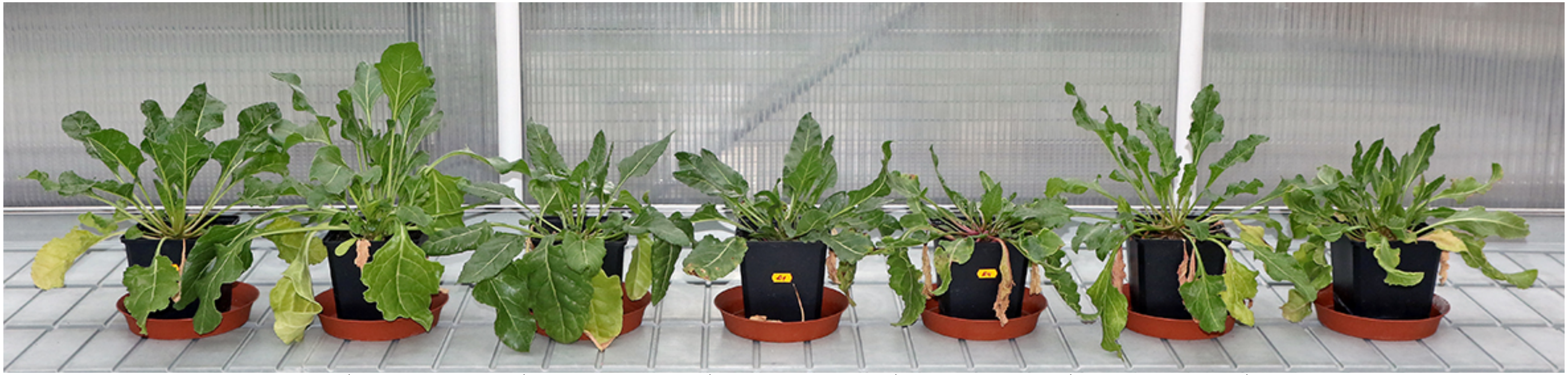

From left to right: control, 44 mmol L^{-1} NaCl, 88 mmol L^{-1} NaCl, 217 mmol L^{-1} NaCl, 434 mmol L^{-1} NaCl, 44 mmol L^{-1} KCl, 434 mmol L^{-1} KCl.

Figure S4. Typical *Cochlearia officinalis* plants 3 weeks after the final treatment with NaCl (A) and KCl (B).

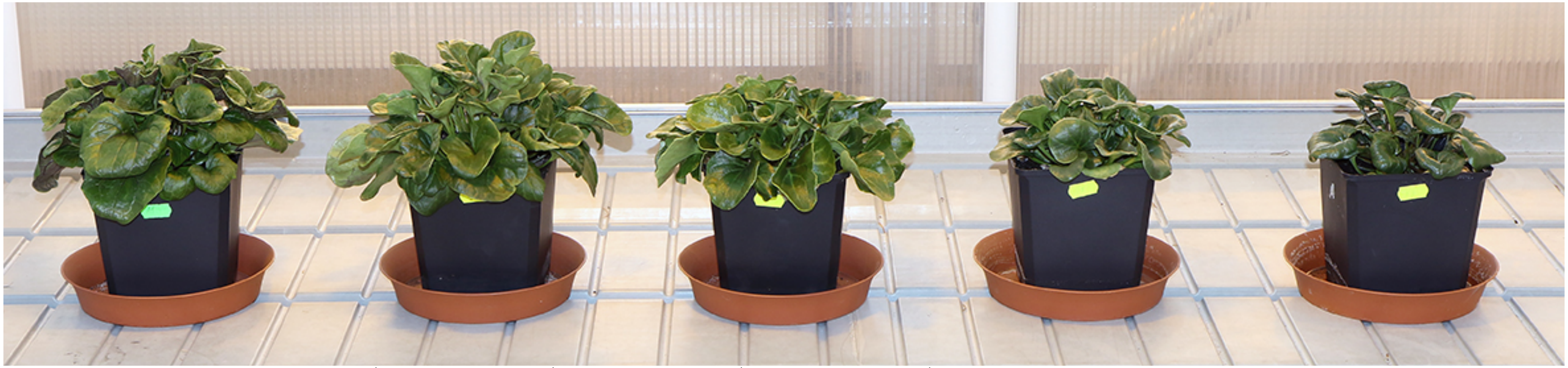

A, from left to right: control, 44 mmol L^{-1} NaCl, 88 mmol L^{-1} NaCl, 217 mmol L^{-1} NaCl, 434 mmol L^{-1} NaCl.

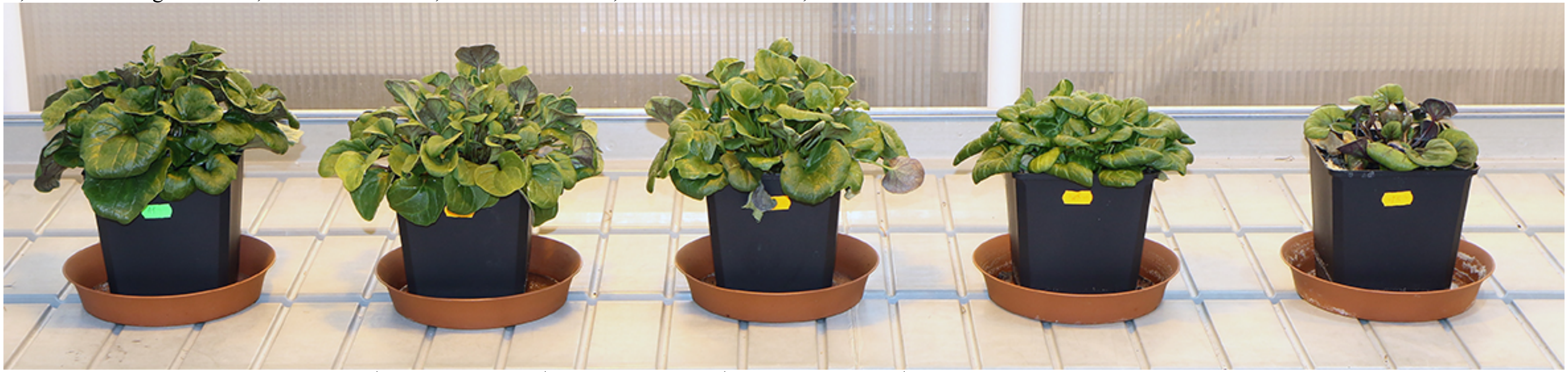

B, from left to right: control, 44 mmol L^{-1} KCl, 88 mmol L^{-1} KCl, 217 mmol L^{-1} KCl, 434 mmol L^{-1} KCl.

Table S1. Effect of NaCl and KCl treatment on morphological parameters of *Plantago maritima* plants. Data are means from 5 replicates ± SE.

**Parameter**	**Control**	**NaCl (mmol L^{-1})**					**KCl (mmol L^{-1})**				
		**22**	**44**	**88**	**217**	**434**	**22**	**44**	**88**	**217**	**434**
Senescent leaves (*n*)	43 ± 17	33 ± 10	22 ± 2	35 ± 6	28 ± 7	28 ± 6	23 ± 3	30 ± 5	27 ± 2	33 ± 8	24 ± 2
Old leaves (*n*)	41 ± 12	42 ± 5	29 ± 3	43 ± 13	40 ± 9	38 ± 4	42 ± 8	33 ± 4	49 ± 13	33 ± 2	8 ± 3
Middle leaves (*n*)	39 ± 5	72 ± 17	57 ± 9	43 ± 12	53 ± 7	60 ± 9	81 ± 10	56 ± 13	97 ± 20	60 ± 11	24 ± 7
Young leaves (*n*)	40 ± 9	70 ± 9	108 ± 25	61 ± 18	94 ± 21	38 ± 6	86 ± 14	64 ± 12	105 ± 31	33 ± 5	69 ± 14
Leaves total (*n*)	164 ± 29	216 ± 23	216 ± 34	182 ± 31	215 ± 25	164 ± 14	232 ± 21	184 ± 21	278 ± 45	158 ± 21	126 ± 22
Flower stalks (*n*)	22 ± 2	28 ± 3	29 ± 4	32 ± 5	26 ± 3	15 ± 2	21 ± 5	30 ± 6	24 ± 6	23 ± 5	4 ± 2
Total length of flower stalks (m)	4.9 ± 0.9	6.0 ± 0.9	6.0 ± 0.6	6.0 ± 0.9	3.4 ± 0.5	1.5 ± 0.4	5.5 ± 2.0	6.8 ± 1.1	4.4 ± 1.2	3.1 ± 0.7	0.5 ± 0.2
DM of senescent leaves (g)	1.9 ± 0.5	1.7 ± 0.2	1.3 ± 0.1	1.6 ± 0.4	1.1 ± 0.2	0.9 ± 0.2	1.5 ± 0.5	2.0 ± 0.3	1.1 ± 0.2	1.5 ± 0.4	1.6 ± 0.2
DM of old leaves (g)	2.3 ± 0.5	2.7 ± 0.1	2.2 ± 0.2	2.6 ± 0.5	1.7 ± 0.3	0.9 ± 0.2	3.1 ± 0.7	2.4 ± 0.3	2.2 ± 0.5	1.7 ± 0.2	0.6 ± 0.2
DM of middle leaves (g)	2.4 ± 0.2	3.8 ± 0.9	3.0 ± 0.6	2.0 ± 0.6	2.0 ± 0.4	1.4 ± 0.2	4.5 ± 0.8	3.6 ± 1.3	4.4 ± 0.5	2.7 ± 0.6	1.0 ± 0.2
DM of young leaves (g)	0.74 ± 0.17	1.36 ± 0.16	2.22 ± 0.45	0.76 ± 0.14	1.01 ± 0.29	0.25 ± 0.06	1.84 ± 0.40	1.06 ± 0.18	1.64 ± 0.43	0.31 ± 0.03	0.94 ± 0.28
DM of leaves total (g)	7.3 ± 0.8	9.5 ± 1.0	8.8 ± 1.1	7.0 ± 1.1	5.8 ± 0.7	3.4 ± 0.4	10.9 ± 1.7	9.1 ± 1.7	9.3 ± 0.6	6.1 ± 1.0	4.1 ± 0.7
DM of flower stalks (g)	1.45 ± 0.39	1.77 ± 0.24	2.00 ± 0.22	1.68 ± 0.17	0.78 ± 0.13	0.37 ± 0.11	1.48 ± 0.39	2.10 ± 0.26	1.21 ± 0.31	0.84 ± 0.17	0.12 ± 0.05
DM of flowers (g)	1.10 ± 0.28	1.27 ± 0.19	1.84 ± 0.20	1.70 ± 0.25	0.77 ± 0.11	0.55 ± 0.18	1.17 ± 0.42	2.11 ± 0.37	0.90 ± 0.24	1.12 ± 0.22	0.17 ± 0.08
DM of roots (g)	2.4 ± 0.3	3.6 ± 0.4	4.4 ± 1.3	2.4 ± 0.6	2.7 ± 0.3	1.6 ± 0.2	5.8 ± 0.4	4.4 ± 0.9	5.7 ± 0.5	3.1 ± 0.5	1.7 ± 0.4

Table S2. Effect of NaCl and KCl treatment on morphological parameters of *Beta vulgaris* subsp. *vulgaris* var. *cicla* plants. Data are means from 5 replicates ± SE.

**Parameter**	**Control**	**NaCl (mmol L^{-1})**				**KCl (mmol L^{-1})**			
		**44**	**87**	**217**	**434**	**44**	**87**	**217**	**434**
Senescent leaves (*n*)	3.5 ± 0.3	2.8 ± 0.3	3.6 ± 0.7	3.0 ± 0.5	4.0 ± 0.6	3.7 ± 0.9	3.2 ± 0.2	2.6 ± 0.4	3.4 ± 0.3
Old leaves (*n*)	3.8 ± 0.5	4.5 ± 0.3	3.8 ± 0.2	3.8 ± 0.5	3.2 ± 0.2	4.0 ± 0.0	4.0 ± 0.0	4.0 ± 0.3	3.2 ± 0.4
Middle leaves (*n*)	5.0 ± 1.1	5.5 ± 0.5	5.8 ± 1.2	4.5 ± 0.3	3.4 ± 0.4	5.0 ± 0.6	6.4 ± 0.3	6.4 ± 0.6	2.6 ± 0.4
Young leaves (*n*)	6.3 ± 0.3	9.0 ± 1.4	8.4 ± 1.6	7.8 ± 1.1	8.6 ± 1.6	8.0 ± 1.2	7.4 ± 0.8	8.2 ± 0.9	7.0 ± 0.6
Leaves total (*n*)	18.5 ± 1.7	21.8 ± 1.7	21.6 ± 2.8	19.0 ± 2.0	19.2 ± 1.5	20.7 ± 0.9	21.0 ± 0.9	21.2 ± 1.2	16.2 ± 0.9
DM of senescent leaf petioles (g)	0.22 ± 0.02	0.31 ± 0.06	0.47 ± 0.18	0.28 ± 0.08	0.36 ± 0.11	0.55 ± 0.16	0.33 ± 0.06	0.32 ± 0.04	0.37 ± 0.06
DM of senescent leaf blades (g)	0.61 ± 0.06	1.00 ± 0.17	1.24 ± 0.21	0.74 ± 0.22	1.18 ± 0.30	1.99 ± 0.65	1.20 ± 0.16	1.32 ± 0.22	1.26 ± 0.23
DM of old leaf petioles (g)	1.43 ± 0.39	1.95 ± 0.27	1.66 ± 0.29	1.07 ± 0.12	0.97 ± 0.20	1.83 ± 0.60	1.50 ± 0.32	1.48 ± 0.31	0.84 ± 0.12
DM of old leaf blades (g)	2.2 ± 0.4	4.3 ± 0.4	2.8 ± 0.3	3.3 ± 0.6	2.8 ± 0.3	2.4 ± 0.4	3.0 ± 0.6	4.6 ± 0.7	3.7 ± 0.5
DM of middle leaf petioles (g)	1.48 ± 0.29	1.82 ± 0.03	1.68 ± 0.23	1.18 ± 0.20	0.81 ± 0.09	2.17 ± 0.73	1.81 ± 0.20	2.06 ± 0.34	0.57 ± 0.12
DM of middle leaf blades (g)	1.58 ± 0.10	2.25 ± 0.10	1.96 ± 0.27	1.90 ± 0.26	1.51 ± 0.17	1.79 ± 0.26	2.18 ± 0.26	2.38 ± 0.28	1.50 ± 0.18
DM of young leaf blades (g)	0.74 ± 0.25	0.70 ± 0.23	0.64 ± 0.06	0.55 ± 0.08	0.52 ± 0.08	0.79 ± 0.19	0.65 ± 0.13	0.84 ± 0.10	0.43 ± 0.06
DM of young leaf petioles (g)	0.87 ± 0.11	1.22 ± 0.38	0.97 ± 0.09	0.93 ± 0.18	0.96 ± 0.14	0.94 ± 0.10	1.00 ± 0.11	0.96 ± 0.12	0.86 ± 0.07
DM of leaf petioles total (g)	3.9 ± 0.7	4.8 ± 0.4	4.4 ± 0.7	3.1 ± 0.1	2.7 ± 0.4	5.4 ± 1.4	4.3 ± 0.5	4.7 ± 0.7	2.2 ± 0.3
DM of leaf blades total (g)	5.2 ± 0.5	8.7 ± 0.7	6.9 ± 0.7	6.9 ± 1.0	6.4 ± 0.6	7.1 ± 1.0	7.4 ± 0.7	9.2 ± 1.1	7.3 ± 0.6
DM of leaves total (g)	9.1 ± 0.9	13.5 ± 1.1	11.4 ± 1.1	9.9 ± 1.0	9.1 ± 0.9	12.5 ± 2.2	11.7 ± 1.2	13.9 ± 1.7	9.5 ± 0.7
DM of roots (g)	5.5 ± 1.4	6.5 ± 1.0	5.5 ± 0.7	3.9 ± 0.8	3.1 ± 0.3	5.0 ± 1.2	6.0 ± 0.8	4.1 ± 0.3	2.9 ± 0.3

Table S3. Effect of NaCl and KCl treatment on morphological parameters of *Beta vulgaris* subsp. *maritima* plants. Data are means from 5 replicates ± SE.

**Parameter**	**Control**	**NaCl (mmol L^{-1})**				**KCl (mmol L^{-1})**	
		**44**	**87**	**217**	**434**	**44**	**434**
Senescent leaves (*n*)	4.3 ± 0.5	3.8 ± 0.9	3.8 ± 0.5	4.5 ± 0.9	3.6 ± 1.1	4.5 ± 0.5	4.6 ± 1.0
Old leaves (*n*)	7.8 ± 0.8	10.0 ± 1.8	7.5 ± 0.9	6.5 ± 0.9	5.2 ± 0.6	6.8 ± 0.5	4.6 ± 0.6
Middle leaves (*n*)	8.5 ± 1.4	8.5 1.2	9.8 ± 1.4	9.3 ± 1.8	7.0 ± 1.0	7.8 ± 0.3	8.2 ± 1.2
Young leaves (*n*)	4.8 ± 0.3	7.5 ± 0.9	8.3 ± 0.9	12.3 ± 3.1	10.0 ± 2.7	7.5 ± 1.0	7.8 ± 1.7
Small leaves (*n*)	18.3 ± 1.8	26.8 ± 3.5	32.3 ± 4.7	39.3 ± 4.9	10.6 ± 2.9	16.3 ± 4.0	16.0 ± 5.6
Leaves total (*n*)	44 ± 1	57 ± 3	62 ± 7	72 ± 9	45 ± 6	43 ± 4	41 ± 9
DM of senescent leaf petioles (g)	0.19 ± 0.07	0.36 ± 0.10	0.17 ± 0.05	0.33 ± 0.12	0.40 ± 0.10	0.39 ± 0.14	0.33 ± 0.08
DM of senescent leaf blades (g)	0.65 ± 0.18	1.45 ± 0.34	1.29 ± 0.51	1.85 ± 0.53	1.84 ± 0.47	2.32 ± 1.12	1.53 ± 0.25
DM of old leaf petioles (g)	1.21 ± 0.32	2.12 ± 0.24	0.98 ± 0.11	0.71 ± 0.09	0.75 ± 0.07	1.38 ± 0.31	0.64 ± 0.11
DM of old leaf blades (g)	2.5 ± 0.2	4.5 ± 0.4	3.4 ± 0.4	2.9 ± 0.3	2.9 ± 0.4	3.8 ± 0.7	3.5 ± 0.8
DM of middle leaf petioles (g)	1.37 ± 0.15	1.32 ± 0.15	0.95 ± 0.06	0.69 ± 0.08	0.61 ± 0.13	1.36 ± 0.18	0.56 ± 0.08
DM of middle leaf blades (g)	2.17 ± 0.37	2.14 ± 0.20	2.23 ± 0.16	1.68 ± 0.16	1.46 ± 0.15	2.18 ± 0.15	1.86 ± 0.29
DM of young leaf petioles (g)	0.19 ± 0.04	0.22 ±0.04	0.18 ± 0.06	0.32 ± 0.07	0.22 ± 0.04	0.45 ± 0.10	0.20 ± 0.06
DM of young leaf blades (g)	0.48 ± 0.09	0.57 ± 0.10	0.55 ± 0.13	0.74 ± 0.16	0.59 ± 0.06	0.76 ± 0.12	0.54 ± 0.14
DM of small leaf petioles (g)	0.17 ± 0.04	0.41 ± 0.12	0.38 ± 0.15	0.44 ± 0.12	0.19 ± 0.06	0.17 ± 0.05	0.18 ± 0.06
DM of small leaf blades (g)	0.32 ± 0.04	0.85 ± 0.25	1.10 ± 0.43	1.03 ± 0.22	0.48 ± 0.10	0.34 ± 0.10	0.59 ± 0.19
DM of leaf petioles total (g)	3.1 ± 0.4	4.4 ± 0.3	2.7 ± 0.3	2.5 ± 0.2	2.2 ± 0.2	3.7 ± 0.4	2.6 ± 0.3
DM of leaf blades total (g)	6.1 ± 0.4	9.5 ± 0.4	8.6 ± 0.6	7.7 ± 0.5	6.9 ± 0.9	8.3 ± 0.4	7.9 ± 1.2
DM of leaves total (g)	9.2 ± 0.6	13.9 ± 0.5	11.2 ± 0.9	10.2 ± 0.6	9.1 ± 1.0	12.0 ± 0.7	10.5 ± 1.4
DM of roots (g)	6.3 ± 1.0	8.9 ± 1.0	6.2 ± 1.0	4.3 ± 0.6	3.7 ± 0.4	8.1 ± 0.9	2.6 ± 0.3

Table S4. Effect of NaCl and KCl treatment on morphological parameters of *Cochlearia officinalis* plants. Data are means from 5 replicates ± SE.

**Parameter**	**Control**	**NaCl (mmol L^{-1})**				**KCl (mmol L^{-1})**			
		**44**	**87**	**217**	**434**	**44**	**87**	**217**	**434**
Leaves (*n*)	112 ± 15	114 ± 27	108 ± 9	63 ± 8	45 ± 11	110 ± 9	127 ± 12	76 ± 13	52 ± 4
DM of leaf petioles (g)	3.8 ± 0.6	3.4 ± 0.4	2.6 ± 0.1	1.7 ± 0.1	1.1 ± 0.1	3.3 ± 0.1	3.0 ± 0.2	1.5 ± 0.2	1.0 ± 0.1
DM of leaf blades (g)	11.5 ± 1.0	15.2 ± 1.1	12.1 ±0.8	7.7 ± 0.9	5.7 ± 0.3	13.5 ± 0.1	14.5 ± 0.9	7.6 ± 0.7	5.1 ± 0.6
DM of leaves (g)	15.3 ± 1.2	18.7 ± 1.0	14.7 ± 0.8	9.4 ± 1.0	6.8 ± 0.4	16.8 ± 0.1	17.5 ± 0.9	9.1 ± 0.8	6.1 ± 0.7
DM of roots (g)	2.58 ± 0.49	1.36 ± 0.26	1.79 ± 0.32	0.97 ± 0.10	0.49 ± 0.03	2.29 ± 0.36	1.54 ± 0.14	0.82 ± 0.09	0.20 ± 0.05

Table S5. Effect of NaCl and KCl treatment on morphological parameters of *Mentha aquatica* plants. Data are means from 3 replicates ± SE, each with 3 plants.

**Parameter**	**Control**	**NaCl (mmol L^{-1})**				**KCl (mmol L^{-1})**			
		**25**	**50**	**100**	**200**	**25**	**50**	**100**	**200**
Leaves (*n*)	160 ± 16	79 ± 24	31 ± 6	16 ± 3	13 ± 2	91 ± 18	76 ± 8	52 ± 09	25 ± 3
Total length of stems (cm)	84 ± 6	70 ± 11	37 ± 2	36 ± 6	30 ± 4	51 ± 11	55 ± 4	45 ± 6	43 ± 7
DM of stems (g)	0.80 ± 0.12	0.49 ± 0.03	0.43 ± 0.15	0.34 ± 0.09	0.29 ± 0.04	0.41 ± 0.18	0.64 ± 0.13	0.51 ± 0.16	0.42 ± 0.08
DM of leaves (g)	0.90 ± 0.14	0.73 ± 0.10	0.25 ± 0.02	0.18 ± 0.03	0.21 ± 0.05	0.52 ± 0.02	0.44 ± 0.06	0.36 ± 0.05	0.19 ± 0.05
DM of roots (g)	0.12 ± 0.02	0.08 ± 0.01	0.10 ± 0.03	0.05 ± 0.02	0.08 ± 0.03	0.09 ± 0.01	0.08 ± 0.01	0.08 ± 0.01	0.05 ± 0.01

Table S6. Effect of NaCl and KCl treatment on Na^+ and K^+ concentration and electrical conductivity (EC) in different parts of *Beta vulgaris* subsp. *vulgaris* var. *cicla* plants. Data are means from 5 replicates ± SE.

Parameter	Plant part	Control	NaCl (mmol L^{-1})				KCl (mmol L^{-1})			
			44	87	217	434	44	87	217	434
Na^+ (g kg^{-1} DM)	Senescent leaf petioles	7.6 ± 1.0	48.4 ± 7.5	52.0 ± 3.9	48.0 ± 12.3	85.0 ± 14.4	4.2 ± 0.3	3.1 ± 1.4	1.5 ± 0.2	1.6 ± 0.1
	Senescent leaf blades	11.6 ± 1.8	60.6 ± 7.7	80.0 ± 3.9	73.1 ± 16.7	108.9 ± 17.3	8.8 ± 1.9	5.7 ± 0.9	4.5 ± 0.2	4.7 ± 0.3
	Old leaf petioles	6.2 ± 0.7	41.1 ± 7.6	46.6 ± 4.1	68.1 ± 5.1	83.3 ± 4.4	3.0 ± 0.7	2.1 ± 0.2	1.9 ± 0.2	1.7 ± 0.1
	Old leaf blades	11.1 ± 2.8	58.3 ± 2.6	69.5 ± 2.4	95.0 ± 9.8	119.0 ± 4.7	8.3 ± 0.9	6.3 ± 0.2	5.3 ± 1.1	4.9 ± 0.3
	Middle leaf petioles	4.6 ± 0.6	35.1 ± 5.0	30.7 ± 4.0	50.5 ± 2.5	73.8 ± 3.1	2.7 ± 0.3	1.4 ± 0.1	1.1 ± 0.1	1.0 ± 0.1
	Middle leaf blades	12.3 ± 2.3	37.9 ± 11.3	43.7 ± 3.7	68.3 ± 7.3	86.2 ± 3.9	7.6 ± 1.4	4.5 ± 0.1	3.4 ± 0.5	3.0 ± 0.1
	Young leaf petioles	2.8 ± 0.4	17.6 ± 2.9	22.7 ± 2.8	33.8 ± 1.7	48.3 ± 1.6	2.6 ± 0.1	1.7 ± 0.1	1.4 ± 0.1	1.5 ± 0.1
	Young leaf blades	9.3 ± 1.1	31.4 ± 2.5	32.7 ± 3.5	43.2 ± 4.1	60.6 ± 2.4	8.1 ± 1.2	4.9 ± 0.4	3.9 ± 0.4	3.8 ± 0.3
	Fine roots	3.5 ± 0.2	12.7 ± 0.7	16.3 ± 1.3	13.8 ± 1.6	16.5 ± 1.9	2.02 ± 0.38	1.23 ± 0.18	0.83 ± 0.04	0.78 ± 0.04
	Middle roots	2.2 ± 0.5	11.1 ± 1.0	12.6 ± 2.8	12.7 ± 3.0	19.1 ± 0.7	1.12 ± 0.02	0.97 ± 0.04	0.80 ± 0.06	0.75 ± 0.00
	Tap root	1.4 ± 0.3	7.6 ± 1.0	11.2 ± 0.4	7.9 ± 1.7	9.7 ± 1.8	0.82 ± 0.14	0.65 ± 0.03	0.67 ± 0.04	0.75 ± 0.06
K^+ (g kg^{-1} DM)	Senescent leaf petioles	49 ± 8	49 ± 12	37 ± 12	38 ± 10	35 ± 3	147 ± 6	158 ± 32	156 ± 15	137 ± 4
	Senescent leaf blades	52 ± 10	32 ± 5	24 ± 9	30 ± 2	29 ± 1	144 ± 15	152 ± 14	177 ± 19	173 ± 7
	Old leaf petioles	14 ± 5	18 ± 8	21 ± 3	19 ± 9	20 ± 5	106 ± 22	144 ± 23	178 ± 5	195 ± 8
	Old leaf blades	36 ± 2	28 ± 1	24 ± 6	25 ± 2	19 ± 2	116 ± 8	140 ± 5	201 ± 7	173 ± 3
	Middle leaf petioles	13 ± 3	14 ± 6	20 ± 3	23 ± 9	26 ± 5	65 ± 15	104 ± 10	128 ± 10	175 ± 12
	Middle leaf blades	31 ± 2	20 ± 1	24 ± 3	26 ± 3	18 ± 1	76 ± 2	106 ± 4	154 ± 23	245 ± 5
	Young leaf petioles	21 ± 1	24 ± 5	30 ± 5	34 ± 7	35 ± 2	60 ± 13	93 ± 3	114 ± 15	165 ± 10
	Young leaf blades	32 ± 1	28 ± 1	29 ± 2	29 ± 1	26 ± 1	55 ± 5	81 ± 4	108 ± 13	196 ± 10
	Fine roots	13.3 ± 0.7	8.8 ± 1.0	9.7 ± 0.3	9.4 ± 2.8	10.5 ± 0.8	32 ± 1	38 ± 2	37 ± 2	35 ± 3
	Middle roots	16.1 ± 1.9	10.8 ± 1.2	9.2 ± 2.0	9.1 ± 2.8	10.5 ± 3.5	34 ± 4	36 ± 2	42 ± 4	49 ± 3
	Tap root	10.7 ± 1.7	5.7 ± 0.2	5.3 ± 0.4	7.4 ± 0.9	6.3 ± 0.9	19 ± 4	24 ± 4	21 ± 5	45 ± 7
EC (mS m^{-1} kg^{-1} DM)	Senescent leaf petioles	196 ± 32	380 ± 13	348 ± 20	323 ± 53	418 ± 39	373 ± 18	408 ± 45	423 ± 34	392 ± 9
	Senescent leaf blades	240 ± 32	375 ± 41	403 ± 48	387 ± 63	455 ± 41	388 ± 41	413 ± 41	487 ± 42	492 ± 15
	Old leaf petioles	90 ± 17	243 ± 38	277 ± 23	368 ± 43	423 ± 4	320 ± 54	387 ± 57	467 ± 6	497 ± 14
	Middle leaf petioles	69 ± 6	193 ± 33	218 ± 26	300 ± 22	390 ± 10	212 ± 42	323 ± 26	375 ± 28	482 ± 31
	Middle leaf blades	158 ± 11	263 ± 4	285 ± 19	378 ± 27	410 ± 18	268 ± 12	352 ± 17	443 ± 46	637 ± 13
	Young leaf petioles	76 ± 3	137 ± 22	172 ± 21	253 ± 13	312 ± 2	193 ± 38	275 ± 3	305 ± 32	403 ± 21
	Young leaf blades	135 ± 5	197 ± 12	202 ± 19	278 ± 16	325 ± 10	198 ± 18	250 ± 9	290 ± 27	465 ± 15
	Fine roots	66 ± 2	93 ± 2	108 ± 3	101 ± 5	115 ± 8	112 ± 3	132 ± 7	125 ± 8	117 ± 6
	Middle roots	68 ± 9	90 ± 7	81 ± 17	92 ± 1	117 ± 13	111 ± 12	115 ± 5	133 ± 10	153 ± 350 ±
	Tap root	50 ± 7	64 ± 5	78 ± 3	74 ± 3	74 ± 4	67 ± 13	86 ± 8	77 ± 13	148 ± 20

Table S7. Effect of NaCl and KCl treatment on Na^+ and K^+ concentration and electrical conductivity (EC) in different parts of *Beta vulgaris* subsp. *maritima* plants. Data are means from 5 replicates ± SE.

**Parameter**	**Plant part**	**Control**	**NaCl (mmol L^{-1})**				**KCl (mmol L^{-1})**	
			**44**	**87**	**217**	**434**	**44**	**434**
Na^+ (g kg^{-1} DM)	Senescent leaf petioles	6.0 ± 0.9	47.7 ± 4.9	54.9 ± 3.9	62.4 ± 9.9	85.4 ± 10.1	1.98 ± 0.30	1.89 ± 0.33
	Senescent leaf blades	8.5 ± 0.6	62.3 ± 7.9	58.1 ± 5.9	90.0 ± 10.5	93.0 ± 16.4	4.55 ± 0.25	3.74 ± 0.70
	Old leaf petioles	6.9 ± 2.0	40.5 ± 3.5	52.5 ± 1.4	61.0 ± 2.5	72.7 ± 7.8	1.63 ± 0.15	1.46 ± 0.17
	Old leaf blades	8.0 ± 1.3	53.1 ± 8.3	54.6 ± 5.6	90.0 ± 13.9	90.0 ± 13.1	4.38 ± 0.58	3.26 ± 0.52
	Middle leaf petioles	6.0 ± 1.9	24.8 ± 3.0	41.4 ± 0.7	51.8 ± 2.0	57.9 ± 8.7	1.55 ± 0.28	1.45 ± 0.15
	Middle leaf blades	8.8 ± 1.6	30.0 ± 3.7	44.1 ± 6.3	59.8 ± 4.3	63.4 ± 10.3	4.52 ± 0.78	2.41 ± 0.40
	Young leaf petioles	4.7 ± 0.9	18.9 ± 0.6	24.3 ± 2.7	36.2 ± 4.5	43.8 ± 5.7	1.32 ± 0.22	2.03 ± 0.66
	Young leaf blades	6.3 ± 0.6	18.2 ± 1.0	22.8 ± 2.5	35.3 ± 5.0	40.5 ± 5.6	3.48 ± 0.57	1.85 ± 0.33
	Small leaf petioles	9.2 ± 2.6	35.1 ± 3.4	49.8 ± 3.7	55.4 ± 4.7	64.5 ± 8.8	1.85 ± 0.30	1.59 ± 0.07
	Small leaf blades	9.0 ± 1.3	43.6 ± 2.0	60.1 ± 8.5	60.6 ± 5.8	70.0 ± 8.8	4.62 ± 0.61	2.65 ± 0.41
	Fine roots	1.5 ± 0.4	5.6 ± 0.9	10.2 ± 0.6	9.0 ± 0.5	9.5 ± 2.0	0.98 ± 0.20	0.73 ± 0.04
	Middle roots	1.6 ± 0.4	3.7 ± 0.9	6.5 ± 0.6	8.8 ± 2.8	13.0 ± 3.8	0.60 ± 0.08	0.92 ± 0.09
	Tap root	1.2 ± 0.2	2.6 ± 0.4	4.6 ± 0.4	4.1 ± 0.3	9.8 ± 2.3	0.47 ± 0.04	0.83 ± 0.12
K^+ (g kg^{-1} DM)	Senescent leaf petioles	22.0 ± 3.8	29.2 ± 12.5	12.0 ± 2.1	19.1 ± 3/0	16.9 ± 4.7	66 ± 11	139 ± 23
	Senescent leaf blades	26.5 ± 5.6	22.6 ± 10.7	11.9 ± 1.9	17.2 ± 4.1	11.0 ± 2.6	75 ± 10	155 ± 24
	Old leaf petioles	8.3 ± 1.9	14.2 ± 4.9	11.6 ± 2.7	23.4 ± 5.2	11.9 ± 2.6	68 ± 10	158 ± 18
	Old leaf blades	13.7 ± 2.7	13.7 ± 4.9	11.4 ± 2.3	17.5 ± 2.1	10.5 ± 1.5	72 ± 6	198 ± 19
	Middle leaf petioles	11.8 ± 1.0	14.9 ± 3.8	11.4 ± 1.1	26.4 ± 3.9	11.3 ± 2.2	53 ± 10	150 ± 18
	Middle leaf blades	14.5 ± 1.0	15.4 ± 4.4	11.1 ± 1.1	24.6 ± 3.5	12.0 ± 1.3	53 ± 7	168 ± 25
	Young leaf petioles	16.8 ± 2.2	24.7 ± 5.1	17.0 ± 1.1	32.9 ± 6.4	14.2 ± 1.7	39 ± 6	115 ± 13
	Young leaf blades	16.2 ± 1.2	21.7 ± 4.7	14.9 ± 1.2	28.9 ± 5.0	15.0 ± 1.4	37 ± 5	115 ± 9
	Small leaf petioles	16.0 ± 2.3	16.7 ± 4.2	11.4 ± 0.7	33.9 ± 3.4	13.6 ± 1.6	68 ± 6	148 ± 8
	Small leaf blades	12.8 ± 1.4	15.6 ± 3.3	10.1 ± 0.6	28.9 ± 3.8	11.5 ± 1.4	68 ± 8	181 ± 20
	Fine roots	21.8 ± 5.6	13.8 ± 2.9	12.6 ± 2.8	2.4 ± 0.5	2.4 ± 0.3	27 ± 9	51 ± 11
	Middle roots	17.8 ± 1.0	25.8 ± 11.8	14.7 ± 2.5	5.1 ± 0.7	5.0 ± 1.0	24 ± 8	89 ± 22
	Tap root	12.3 ± 1.5	17.4 ± 6.9	11.6 ± 0.5	4.1 ± 0.7	5.0 ± 0.8	14 ± 5	89 ± 24
EC (mS m^{-1} kg^{-1} DM)	Senescent leaf petioles	104 ± 17	279 ± 21	299 ± 26	276 ± 17	424 ± 48	282 ± 48	488 ± 46
	Senescent leaf blades	114 ± 17	322 ± 19	308 ± 21	382 ± 22	440 ± 25	328 ± 33	553 ± 61
	Old leaf petioles	68 ± 11	224 ± 16	291 ± 7	274 ± 31	375 ± 20	282 ± 34	534 ± 36
	Middle leaf petioles	72 ± 7	165 ± 16	251 ± 8	255 ± 15	318 ± 29	212 ± 35	444 ± 46
	Middle leaf blades	98 ± 11	189 ± 12	255 ± 22	268 ± 15	339 ± 22	228 ± 22	530 ± 51
	Young leaf petioles	84 ± 11	163 ± 6	203 ± 10	223 ± 7	277 ± 18	155 ± 17	393 ± 34
	Young leaf blades	93 ± 6	155 ± 7	190 ± 5	211 ± 6	265 ± 12	160 ± 13	388 ± 22
	Small leaf petioles	101 ± 15	204 ± 14	273 ± 14	267 ± 19	330 ± 22	272 ± 22	511 ± 38
	Small leaf blades	90 ± 10	238 ± 14	311 ± 29	277 ± 16	354 ± 25	282 ± 14	571 ± 28
	Fine roots	38 ± 9	42 ± 4	63 ± 2	67 ± 3	69 ± 9	78 ± 8	120 ± 8

	Middle roots	32 ± 1	38 ± 4	53 ± 5	97 ± 19	91 ± 16	62 ± 6	203 ± 18
	Tap root	21 ± 1	29 ± 3	41 ± 3	50 ± 5	81 ± 12	38 ± 5	200 ± 23

Table S8. Effect of NaCl and KCl treatment on Na^+ and K^+ concentration and electrical conductivity (EC) in different parts of *Cochlearia officinalis* plants. Data are means from 5 replicates ± SE.

**Parameter**	**Plant part**	**Control**	**NaCl (mmol L^{-1})**				**KCl (mmol L^{-1})**			
			**44**	**87**	**217**	**434**	**44**	**87**	**217**	**434**
Na^+ (g kg^{-1} DM)	Leaf petioles	2.7 ± 0.4	24.5 ± 1.8	29.6 ± 3.8	38.5 ± 7.1	43.7 ± 5.8	1.14 ± 0.03	1.08 ± 0.09	1.43 ± 0.13	1.95 ± 0.15
	Leaf blades	2.9 ± 0.5	24.9 ± 1.8	35.8 ± 8.1	49.5 ± 4.1	52.6 ± 5.1	2.01 ± 0.06	1.81 ± 0.37	2.07 ± 0.16	2.36 ± 0.27
	Roots	3.8 ± 1.6	10.8 ± 1.9	12.0 ± 0.7	8.5 ± 1.2	9.5 ± 1.8	0.58 ± 0.04	0.77 ± 0.04	0.68 ± 0.03	0.99 ± 0.09
K^+ (g kg^{-1} DM)	Leaf petioles	13.2 ± 4.8	17.5 ± 2.3	27.1 ± 2.3	26.1 ± 5.2	33.0 ± 6.7	75.8 ± 3.0	80.8 ± 3.0	140.0 ± 7.6	215.8 ± 8.5
	Leaf blades	8.0 ± 2.4	12.2 ± 3.6	8.7 ± 0.4	7.7 ± 1.0	12.2 ± 1.6	54.2 ± 3.0	50.8 ± 4.8	100.0 ± 15.1	172.3 ± 26.2
	Roots	6.4 ± 1.8	5.6 ± 1.2	6.8 ± 0.7	12.0 ± 2.3	9.6 ± 1.4	18.3 ± 2.2	31.3 ±1.6	31.4 ± 2.2	48.9 ± 11.9
EC (mS m^{-1} kg^{-1} DM)	Leaf petioles	97 ± 19	172 ± 27	255 ± 15	280 ± 38	298 ± 36	238 ± 11	261 ± 6	387 ± 17	572 ± 25
	Roots	52 ± 6	85 ± 10	90 ± 3	92 ± 10	105 ± 15	75 ± 9	115 ± 5	110 ± 9	165 ± 30

Table S9. Effect of NaCl and KCl treatment on Na^+ and K^+ concentration and electrical conductivity (EC) in different parts of *Mentha aquatica* plants. Data are means from 5 replicates ± SE.

Parameter	Plant part	Control	NaCl (mmol L^{-1})				KCl (mmol L^{-1})			
			**25**	**50**	**100**	**200**	**25**	**50**	**100**	**200**
Na^+ (g kg^{-1} DM)	Leaves	2.3 ± 0.1	21.8 ± 2.3	33.0 ± 0.8	40.5 ± 2.5	35.8 ± 4.2	0.73 ± 0.05	0.68 ± 0.01	0.96 ± 0.04	0.93 ± 0.07
	Stems	2.5 ± 0.2	14.3 ± 0.8	28.1 ± 2.7	28.9 ± 2.7	86.0 ± 8.5	0.84 ± 0.04	1.08 ± 0.17	0.99 ± 0.04	1.73 ± 0.02
	Roots	6.5 ± 0.5	13.6 ± 0.9	15.2 ± 1.4	17.5 ± 0.3	10.2 ± 1.0	1.25 ± 0.05	0.92 ± 0.10	0.78 ± 0.08	1.11 ± 0.05
K^+ (g kg^{-1} DM)	Leaves	52 ± 2	33 ± 4	15 ± 1	14 ± 1	12 ± 1	58 ± 3	63 ± 2	144 ± 8	143 ± 14
	Stems	87 ± 2	61 ± 4	40 ± 2	40 ± 3	30 ± 2	78 ± 3	82 ± 9	88 ± 9	131 ± 4
	Roots	54 ± 3	46 ± 4	30 ± 5	9 ± 2	3 ± 1	62 ± 2	61 ± 2	38 ± 3	25 ± 4
EC (mS m^{-1} kg^{-1} DM)	Stems	164 ± 2	167 ± 6	163 ± 12	164 ± 12	328 ± 14	232 ± 11	252 ± 23	228 ± 27	378 ± 8
	Roots	80 ± 4	102 ± 9	107 ± 13	86 ± 8	38 ± 5	166 ± 6	187 ± 7	92 ± 12	52 ± 4
